# Plant Proteins and Bioactive Peptides: Distinguishing Nutritional Quality from Sequence-Dependent Bioactivity

**DOI:** 10.3390/biotech15040089

**Published:** 2026-10-07

**Authors:** Tatevik Sargsyan, Heghine Hakobyan, Hasmik Grigoryan, Lala Stepanyan, Anahit Hovhannisyan, Zhaklina Saribekyan, Avetis Tsaturyan, Nelli Hovhannisyan, Ashot Saghyan, Caterina Vicidomini, Rosanna Palumbo, Giovanni N. Roviello

**Affiliations:** 1 Scientific and Production Center “Armbiotechnology” RA, 14 Gyurjyan Str., Yerevan 0056, Armenia; 2 Institute of Pharmacy, Yerevan State University, 1 Alex Manoogian Str., Yerevan 0025, Armenia; 3 Institute of Biostructures and Bioimaging (IBB), Italian National Council for Research (CNR), Area di Ricerca Site and Headquarters, Via Pietro Castellino 111, 80131 Naples, Italy

**Keywords:** plant peptides, bioactive proteins, essential amino acids, plant biotechnology, therapeutic applications, protein hydrolysates, functional foods, nutraceuticals, antioxidant activity, antimicrobial peptides, molecular docking, artificial intelligence, bioactive compounds

## Abstract

Plant proteins represent both important nutritional resources and reservoirs of encrypted bioactive peptide sequences. However, protein nutritional quality and peptide bioactivity are distinct properties: a favorable indispensable amino acid profile or high digestibility does not necessarily predict the generation of biologically active peptides. This review examines plant-derived proteins and peptides with particular emphasis on the relationship between parent-protein composition, peptide release, sequence-dependent activity, analytical identification, and biological validation. The relevant peer-reviewed literature was critically evaluated, with detailed search and selection criteria. Major plant protein sources, peptide-generation strategies, purification and mass-spectrometric characterization approaches, and reported antioxidant, antimicrobial, antihypertensive, antidiabetic, anti-inflammatory, and other regulatory activities are discussed. Particular attention is given to experimentally validated peptide sequences and to the hierarchy of evidence from computational prediction and biochemical assays to cellular, animal, and human studies. Structure–activity relationships are interpreted cautiously, recognizing that peptide length, charge, hydrophobicity, and individual amino acid residues are contributory rather than independently predictive features. The review further integrates computational prediction with experimental validation and highlights gastrointestinal stability, intestinal transport, bioavailability, safety, scalability, and regulatory considerations as major translational challenges. This evidence-based framework distinguishes nutritional adequacy from peptide function and supports more rigorous development of plant-derived bioactive peptides for functional-food, nutraceutical, and related applications.

## 1. Introduction

Proteins and peptides derived from plants have gained increasing scientific attention because of their combined nutritional, functional, and biomedical relevance. In addition to serving as dietary sources of essential amino acids, plant proteins contain encrypted peptide sequences that may be released during gastrointestinal digestion, enzymatic hydrolysis, microbial fermentation, germination, or controlled food processing. Once liberated, these peptides can exert diverse biological activities, including antioxidant, antimicrobial, anti-inflammatory, immunomodulatory, antihypertensive, antidiabetic, lipid-regulating, metal-chelating, and anticancer-related effects. These reported activities have prompted interest in plant-derived peptides as candidates for functional food and nutraceutical research. However, activity observed in computational or in vitro studies alone does not establish physiological or therapeutic efficacy [1,2,3].

Plants providing proteins with favorable essential amino acid profiles are of particular interest because their protein composition, structural organization, amino acid sequence, and susceptibility to proteolysis can influence the profile of peptides released during digestion or controlled processing. Legumes, cereals, pseudocereals, oilseeds, and agro-industrial plant by-products provide diverse protein fractions, including albumins, globulins, prolamins, glutelin and storage proteins, which may yield peptides with distinct physicochemical and biological properties. The biological activity of these peptides depends on the combined effects of peptide sequence, residue position, length, hydrophobicity, net charge, amphipathicity, conformation, proteolytic stability, and accessibility to biological targets. However, the translation of in vitro peptide activity into physiological relevance remains challenging because peptide stability, intestinal absorption, bioavailability, toxicity, allergenicity, and in vivo efficacy are not always systematically evaluated [1,2,4,5].

Recent advances in plant biotechnology, green extraction technologies, enzymatic and microbial hydrolysis, membrane-based fractionation, chromatographic purification, and high-resolution mass spectrometry have greatly expanded the ability to isolate and characterize plant-derived bioactive peptides. In parallel, peptidomics, molecular docking, molecular dynamics simulations, machine learning, and bioinformatics-based prediction tools have accelerated the identification of candidate peptides and their potential biological targets. The integration of experimental and computational approaches now enables a more rational workflow for discovering plant peptides with defined mechanisms of action, improved reproducibility, and potential translational value [6,7,8,9,10].

This review focuses on the relationship between parent-protein composition and nutritional quality, peptide generation, peptide sequence and structural characteristics, and experimentally demonstrated biological activity. Particular attention is given to how protein structure and processing conditions influence peptide release, how sequence-dependent physicochemical properties affect biological function, and how computational predictions should be integrated with biochemical, cellular, and in vivo validation. This framework provides a more focused progression from plant protein composition to peptide generation, biological function, and translational relevance.

Recent reviews have summarized the sources, production methods, and reported activities of plant-derived bioactive peptides [11,12,13]. The present review focuses on how the nutritional quality and structure of parent proteins relate to peptide generation, and on the analytical and experimental evidence needed to attribute biological activity to an identified peptide.

## 2. Review Methodology

This review was based on a structured literature search using PubMed, Scopus, Web of Science, and Google Scholar, among other sources. The search primarily focused on the peer-reviewed literature published between 2021 and September 2026. Earlier publications were considered only when they represented seminal methodological studies, internationally recognized standards, or original primary evidence that could not be adequately replaced by the more recent literature.

Search terms included combinations of “plant-derived bioactive peptides”, “plant proteins”, “plant protein hydrolysates”, “essential amino acids”, “protein quality”, “bioactive peptide identification”, “LC–MS/MS”, “antioxidant peptides”, “antimicrobial peptides”, “ACE-inhibitory peptides”, “antidiabetic peptides”, “gastrointestinal stability”, “intestinal transport”, “bioavailability”, “molecular docking”, and “machine learning”.

Priority was given to peer-reviewed studies reporting plant-derived proteins or peptides, defined peptide sequences, analytical identification, quantitative biological activity, gastrointestinal stability, transport, in vivo or human evidence, or computational approaches linked to experimental validation. Studies unrelated to plant-derived peptides or lacking sufficient experimental detail were excluded.

## 3. Plant Proteins with Favorable Essential Amino Acid Profiles as Sources of Bioactive Peptides

Plant proteins with favorable essential amino acid profiles represent an important reservoir of encrypted bioactive peptide sequences with nutritional, physiological, and biotechnological relevance. Their functional potential is determined not only by total protein content or amino acid composition, but also by protein architecture, digestibility, protease-accessible cleavage sites, and the physicochemical properties of the peptides released during gastrointestinal digestion, fermentation, or controlled enzymatic hydrolysis. Legumes, cereals, pseudocereals, and oilseed plants contain diverse storage and functional proteins that can generate peptides with antioxidant, antihypertensive, antimicrobial, antidiabetic, anti-inflammatory, immunomodulatory, opioid-like, lipid-regulating, and metal-chelating activities. Therefore, plant proteins should be considered not merely as dietary nitrogen sources, but as structured precursors of biologically active molecular fragments [14,15,16,17].

The biological activity of plant-derived peptides depends on sequence context, peptide length, hydrophobicity, charge distribution, amphiphilicity, and resistance to enzymatic degradation. In this context, plants providing proteins with favorable essential amino acid profiles may offer nutritional advantages, while their capacity to generate bioactive peptides should be evaluated independently on the basis of protein sequence, proteolytic accessibility, processing conditions, and the properties of the released peptides. However, the physiological relevance of many reported plant peptides remains dependent on their gastrointestinal stability, intestinal absorption, plasma persistence, and ability to reach molecular targets at biologically meaningful concentrations. Thus, a critical evaluation of plant-derived bioactive peptides requires an integrated view linking botanical source, protein composition, hydrolysis conditions, peptide structure, mechanism of action, and validation model [18,19,20,21,22].

### 3.1. Nutritional Quality, Essential Amino Acid Profiles, and Peptide-Generating Potential

Essential amino acids cannot be synthesized in sufficient amounts by the human body and must therefore be obtained through the diet. Histidine, isoleucine, leucine, lysine, methionine, phenylalanine, threonine, tryptophan, and valine are consequently central to protein nutritional quality and also contribute to the chemical diversity of food-derived bioactive peptides [14,23].

Assessment of protein nutritional quality requires more than consideration of total protein content or the presence of individual essential amino acids. A limiting amino acid is the indispensable amino acid present in the lowest proportion relative to human physiological requirements and can therefore restrict the effective nutritional utilization of the remaining amino acids. Amino acid scoring approaches compare the indispensable amino acid pattern of a dietary protein with an established reference pattern, whereas digestibility determines the proportion of ingested protein or amino acids that becomes biologically available [23,24].

The Protein Digestibility-Corrected Amino Acid Score (PDCAAS) integrates the amino acid score with overall protein digestibility, while the Digestible Indispensable Amino Acid Score (DIAAS) evaluates the ileal digestibility of individual indispensable amino acids and therefore provides a more amino-acid-specific assessment of protein quality. These parameters are useful for evaluating the nutritional adequacy of plant proteins but should not be interpreted as predictors of peptide bioactivity [4,6].

Importantly, protein nutritional quality and peptide bioactivity represent distinct but potentially complementary characteristics. A plant protein with a favorable indispensable amino acid profile, high digestibility, or a high protein-quality score does not necessarily generate strongly bioactive peptides after digestion or controlled hydrolysis. Peptide bioactivity depends instead on the complete primary sequence of the parent protein, the location and accessibility of proteolytic cleavage sites, the protease or processing conditions used, and the sequence, residue position, terminal composition, conformation, stability, and physicochemical properties of the released peptide. Conversely, proteins of moderate nutritional quality may contain encrypted peptide sequences with substantial biological activity. Accordingly, the nutritional quality of the parent protein and the biological activity of its derived peptides should be evaluated independently before their potential complementary value is considered [3,6,23].

Plant proteins differ markedly in their essential amino acid profiles. Cereals are commonly limited in lysine, whereas legumes are generally richer in lysine but may contain comparatively lower levels of sulfur-containing amino acids, particularly methionine. Cysteine, although not classified as an indispensable amino acid, contributes to the total sulfur amino acid content and can partially spare dietary methionine. Pseudocereals and oilseeds often provide complementary amino acid patterns, thereby broadening the range of peptide sequences that may be released during food processing, fermentation, enzymatic hydrolysis, or gastrointestinal digestion [16,17,25].

From a structure–function perspective, different classes of amino acid residues contribute differently to peptide bioactivity. Branched-chain amino acids, including leucine, isoleucine, and valine, increase peptide hydrophobicity and, depending on their position within the sequence and the overall peptide conformation, may influence interactions with lipid membranes and hydrophobic regions of biological targets [14]. Aromatic residues such as phenylalanine, tyrosine, and tryptophan may contribute to radical scavenging through electron or hydrogen-atom donation and may stabilize peptide–target interactions through hydrophobic and π–π interactions [26]. Sulfur-containing residues, particularly methionine and cysteine, may participate in redox reactions and contribute to the antioxidant properties of certain peptides. However, their effects depend strongly on sequence position, solvent accessibility, peptide conformation, and the oxidation system being studied [26,27].

Basic or cationic residues, particularly lysine and arginine, facilitate electrostatic interactions with negatively charged microbial membranes and other anionic biological surfaces. Histidine may contribute to such interactions when its imidazole side chain is protonated; therefore, its contribution is strongly dependent on pH and the local peptide environment [4,28]. Certain proline-rich sequences may exhibit increased resistance to gastrointestinal proteolysis because the cyclic structure of proline restricts backbone conformation and can reduce the accessibility of adjacent peptide bonds to some proteases. However, this resistance is sequence- and enzyme-dependent and should not be generalized to all proline-containing peptides [4,29].

Consequently, the peptide-generating potential of a plant protein cannot be predicted solely from its amino acid composition. Protein folding, aggregation state, disulfide bonding, and processing-induced changes in secondary and tertiary structure can influence the accessibility of proteolytic cleavage sites and, consequently, the resulting peptide profile [14,30]. The same plant protein substrate may therefore generate markedly different peptide mixtures depending on protease specificity, enzyme-to-substrate ratio, hydrolysis time, pH, temperature, pretreatment method, fermentation conditions, and degree of hydrolysis [14,16,19,31]. Accordingly, the biological activity of plant protein hydrolysates should be interpreted together with their production and processing parameters rather than being attributed solely to the botanical origin or bulk amino acid composition of the parent protein [14,16,17].

### 3.2. Legumes and Cereals as Major Precursors of Bioactive Peptides

Legumes represent one of the most extensively studied plant protein groups for the production of bioactive peptides. Soybean, pea, lentil, chickpea, mung bean, faba bean, lupin, and common bean contain abundant storage proteins, mainly 7S vicilin-type globulins and 11S legumin-type globulins, together with albumins and other minor protein fractions. These proteins are generally richer in lysine than cereal proteins, making legumes nutritionally valuable and suitable substrates for the release of peptides with diverse biological activities [14,16].

Soybean proteins, particularly glycinin and β-conglycinin, have been widely investigated as precursors of peptides with antihypertensive, antioxidant, lipid-regulating, and glucose-modulating properties [14,19,32,33,34]. Soybean-derived peptides have demonstrated ACE-inhibitory activity, modulation of cholesterol-related pathways, and inhibition of enzymes involved in glucose metabolism in biochemical, cellular, and animal models [32,33,34]. Lentil protein hydrolysates have shown antioxidant and ACE-inhibitory activities, depending on the protease and hydrolysis conditions [35]. An enzymatic protein hydrolysate from yellow field pea seeds has shown antioxidant, anti-inflammatory, and immunomodulatory properties in the assays reported by Ndiaye et al. [36]. Chickpea protein hydrolysates have shown DPP-IV inhibitory activity, while LC–MS/MS analysis identified candidate peptide sequences without establishing that each identified peptide was individually responsible for the measured activity [37]. In mung bean, a low-molecular-weight (<3 kDa) hydrolysate fraction showed radical-scavenging, Fe^2+^-chelating, and ACE-inhibitory activities; these results characterize the fraction rather than an individually validated peptide [38]. Faba-bean-derived peptides have also shown biological activity in cellular models, together with evidence of gastrointestinal stability and intestinal transport [21]. These activities are commonly associated with short- and medium-length peptides containing hydrophobic, aromatic, acidic, or basic amino acid residues [14,26].

Cereals, including wheat, rice, maize, barley, oats, rye, and sorghum, constitute another major group of plant proteins capable of releasing bioactive peptides following gastrointestinal digestion, fermentation, or controlled enzymatic hydrolysis [14]. Although many cereal proteins are relatively low in lysine, they contain prolamins, glutelins, albumins, and globulins that can generate peptide sequences with distinct structural and biological properties.

Wheat gluten proteins, including gliadins and glutenins, contain repetitive glutamine- and proline-rich domains that influence their susceptibility to proteolysis and the persistence of digestion-resistant peptide fragments. Some of these peptides may exhibit biological activities, whereas specific gliadin-derived sequences can retain immunogenicity and stimulate adverse immune responses in individuals with coeliac disease [4,29]. Therefore, beneficial and adverse gluten-derived peptide activities should be clearly distinguished.

Rice proteins, particularly rice bran proteins and rice glutelins, are recognized sources of antioxidant and ACE-inhibitory peptides. Individual rice-bran-derived peptides have demonstrated radical-scavenging, metal-chelating, cellular antioxidant, and ACE-inhibitory activities [39,40]. Oat bran protein hydrolysates and identified oat peptides have also exhibited antioxidant activity and inhibition of pancreatic lipase and α-amylase in vitro [41]. Barley protein hydrolysates have yielded several experimentally validated ACE-inhibitory peptides, while maize protein hydrolysates have generated peptides with pronounced radical-scavenging and reducing activities [42,43].

Nevertheless, the functional interpretation of cereal-derived peptides requires careful consideration of protein digestibility, gluten-related immunogenicity, processing and hydrolysis conditions, peptide stability, and the distinction between in vitro bioactivity and confirmed physiological efficacy [4,14,44]. These differences in protein content, major protein fractions, peptide-related activities, and allergenic or technological limitations across major legume and cereal sources highlight the diversity and complexity of their functional and technological properties.

### 3.3. Pseudocereals and Oilseed Plants as Complementary Protein Reservoirs

Pseudocereals, including quinoa, amaranth, and buckwheat, are increasingly recognized as valuable sources of high-quality plant proteins. Compared with conventional cereals, they generally exhibit more balanced essential amino acid profiles, particularly higher lysine contents and, depending on the species and cultivar, favorable levels of sulfur-containing and branched-chain amino acids [15,25]. Quinoa proteins consist predominantly of globulin and albumin fractions and show relatively high susceptibility to gastrointestinal proteolysis, making them suitable substrates for the generation of bioactive peptides [45,46,47,48,49]. Quinoa protein hydrolysates have demonstrated antioxidant, ACE-inhibitory, DPP-IV-inhibitory, and anti-inflammatory or immunomodulatory activities in different in vitro and in vivo experimental systems. Amaranth protein hydrolysates can exhibit ACE-inhibitory, antithrombotic, antioxidant, and anti-inflammatory activities [46,47,48,49,50]. Buckwheat proteins and their digestion-derived peptides have been associated with cholesterol-lowering, antioxidant, and anti-inflammatory effects [49,51,52].

Oilseed plants, including sunflower, sesame, flaxseed, pumpkin seed, hemp, chia, rapeseed, and cottonseed, constitute another important reservoir of essential amino acid-containing proteins. These protein fractions are often obtained as by-products after oil extraction, making them highly relevant for sustainable biorefinery, circular economy strategies, and the development of value-added functional ingredients [17]. Sunflower protein hydrolysates have been associated with antioxidant, antihypertensive, and emulsifying properties. Sesame proteins contain sulfur-containing amino acids and may generate antioxidant and ACE-inhibitory peptides. Flaxseed and chia proteins are rich in arginine and branched-chain amino acids, supporting the formation of peptides potentially involved in blood pressure regulation, oxidative stress control, and inflammatory modulation. Hemp seed proteins, mainly edestin and albumin, are readily hydrolyzed and can yield peptides with antioxidant, antihypertensive, and antimicrobial properties [53,54]. The corresponding protein contents, major protein fractions, reported peptide-related activities, allergenic considerations, and principal limitations of these pseudocereal and oilseed sources are compared in Table 1.

The valorization of oilseed protein by-products is particularly important from both nutritional and technological perspectives. These materials can be converted into peptide-rich hydrolysates with improved solubility, emulsifying capacity, foaming properties, and biological activity. Nevertheless, their application in food and pharmaceutical systems requires attention to sensory quality, residual antinutritional compounds, allergenicity, and batch-to-batch variability [17,55,56,57,58].

### 3.4. Structural Determinants, Gastrointestinal Stability, and Bioavailability

The release and biological performance of plant-derived peptides are strongly governed by the structural organization of their parent proteins. Plant storage proteins differ in molecular weight, tertiary and quaternary structure, disulfide bonding, hydrophobic-core exposure, aggregation behavior, and susceptibility to proteolysis [14,16,30,31]. Compact globular proteins may require physical or biotechnological pretreatments, including heating, ultrasound, high-pressure processing, or fermentation, to induce partial unfolding, expose previously buried cleavage sites, and improve their accessibility to digestive or industrial proteases [16,30,59,60]. In 11S legumin-type globulins, acidic and basic polypeptides linked by disulfide bonds may respond differently to enzymatic cleavage, and the resulting peptide profile is strongly influenced by enzyme specificity and hydrolysis conditions [14,31]. By contrast, the repetitive proline- and glutamine-rich regions of cereal prolamins may restrict complete proteolysis and promote the persistence of digestion-resistant peptide fragments [29,44].

Bioavailability remains one of the major challenges in translating the in vitro activity of plant-derived peptides into physiologically relevant effects. To exert systemic activity, peptides must survive gastric and intestinal digestion, resist degradation by brush-border and plasma peptidases, cross the intestinal epithelial barrier, and reach their molecular targets at biologically effective concentrations [20,21,22,61,62,63,64]. Several pathways may contribute to intestinal peptide absorption. Many di- and tripeptides can be transported through peptide transporter 1 (PepT1), whereas other short peptides may cross the intestinal epithelium through paracellular diffusion via tight junctions [20,62]. Selected oligopeptides may also undergo endocytosis or energy-dependent transcytosis, although the contribution of these pathways depends strongly on peptide size, sequence, charge, hydrophobicity, and resistance to cellular peptidases [61,62,63].

Peptide stability is influenced by molecular size, amino acid sequence, terminal residues, net charge, hydrophobicity, secondary structure, and the accessibility of protease-sensitive peptide bonds [14,20,21,61,62]. Proline-containing sequences may exhibit increased resistance to gastrointestinal proteolysis because the conformational constraints imposed by proline can reduce the accessibility of adjacent peptide bonds to certain proteases; however, this effect is sequence- and enzyme-dependent and should not be generalized to all proline-containing peptides [4,29,44]. Consequently, simulated gastrointestinal digestion, brush-border peptidase assays, Caco-2 monolayer transport studies, plasma or serum stability assays, and in vivo pharmacokinetic analyses are required to determine whether a peptide reaches the systemic circulation intact or in the form of an active metabolite [20,21,22,61,62,64]. Without such validation, biological activities observed exclusively in chemical, enzyme-based, or simplified in vitro assays should be interpreted cautiously.

These terms should be distinguished clearly. Gastrointestinal stability describes the ability of a peptide to remain intact, or to generate active metabolites, during gastric and intestinal digestion. Intestinal transport refers specifically to movement across the intestinal epithelial barrier through mechanisms such as PepT1-mediated transport, paracellular diffusion, endocytosis, or transcytosis. Absorption denotes entry of the peptide or its metabolites from the intestinal lumen into the internal biological compartment, whereas systemic bioavailability refers to their subsequent presence in the systemic circulation, with sufficient exposure at peripheral target tissues required for biological activity. Consequently, resistance to gastrointestinal digestion or transport across a Caco-2 monolayer should not, by itself, be interpreted as evidence of systemic bioavailability or physiological efficacy [20,21,22,61,62,63,64].

### 3.5. Technological, Sensory, and Safety-Related Challenges

The incorporation of plant protein hydrolysates and peptide fractions into functional foods, nutraceuticals, or pharmaceutical formulations is influenced by several technological and safety-related factors. Controlled or limited hydrolysis can improve protein solubility, digestibility, emulsifying activity, and foaming capacity; however, these effects depend strongly on the protein source, enzyme specificity, degree of hydrolysis, pH, and processing conditions [17,55,58,65]. Extensive hydrolysis may generate peptides that are too short to form cohesive interfacial films, thereby reducing emulsion or foam stability and, in some cases, producing undesirable sensory attributes. The degree of hydrolysis is therefore a critical parameter that must be optimized according to the intended application [55,58,65].

Bitterness is one of the most common sensory limitations of plant protein hydrolysates. It is frequently associated with the release of peptides containing exposed hydrophobic residues, including leucine, isoleucine, valine, and phenylalanine, although bitterness is also influenced by peptide sequence, molecular weight, terminal residues, enzyme specificity, and degree of hydrolysis [14,66,67]. The use of enzyme preparations with complementary endopeptidase and exopeptidase activities, sequential enzymatic hydrolysis, and appropriate control of hydrolysis conditions can reduce bitterness while retaining desirable functional properties [56,67]. Encapsulation in lipid- or carbohydrate-based carrier systems, including solid lipid microparticles and maltodextrin- or maltodextrin–pectin-based matrices, can further mask unpleasant tastes, protect peptide fractions, and facilitate their incorporation into food products [68,69]. Nevertheless, debittering procedures should be carefully evaluated because further peptide cleavage, separation, or encapsulation may alter peptide composition, biological activity, release characteristics, and bioavailability.

Safety assessment is equally important. Plant protein matrices may contain antinutritional factors, including phytates, tannins, lectins, saponins, and protease inhibitors, which can impair protein digestibility or mineral bioavailability [17,57,70]. Germination, fermentation, thermal processing, and enzymatic treatment can reduce selected antinutritional compounds and improve protein accessibility; however, their effectiveness depends strongly on the plant matrix, cultivar, microbial strain, treatment intensity, and processing conditions [57,70]. Consequently, the reduction in antinutritional factors should be verified experimentally rather than assumed from the application of a particular processing method.

Hydrolysis may also modify the allergenic potential of plant proteins. Extensive enzymatic hydrolysis can disrupt conformational and linear IgE-binding epitopes and substantially reduce the immunoreactivity of some legume proteins [71,72]. However, hydrolysis should not automatically be assumed to eliminate allergenicity, because residual IgE-reactive fragments may persist, while peptide aggregation or rearrangement may produce multivalent structures capable of retaining immunoreactivity, as demonstrated for some hydrolyzed wheat protein preparations [73]. The allergenic outcome depends on the botanical source, enzyme system, degree of hydrolysis, peptide-size distribution, processing conditions, and the biological model used for evaluation [71,72,73]. Therefore, allergenicity assessment should combine protein and peptide profiling with IgE-binding assays and, where appropriate, basophil-activation, histamine-release, cellular, and in vivo models. Cytotoxicity, genotoxicity, dose–response relationships, microbiological quality, and potential interactions with formulation components should also be evaluated before plant-derived peptide preparations are incorporated into food, nutraceutical, or therapeutic products.

Overall, essential amino acid-rich plant proteins represent a scientifically and technologically promising platform for the generation of bioactive peptides. Their successful application depends on the coordinated interaction among botanical source, protein structure, amino acid composition, extraction and hydrolysis conditions, peptide sequence, sensory characteristics, gastrointestinal stability, bioavailability, safety, and biological target. Future studies should move beyond simple chemical or enzyme-based in vitro screening and adopt standardized workflows combining proteomics and peptidomics, simulated gastrointestinal digestion, intestinal transport models, molecular docking, machine-learning-assisted prediction, toxicological assessment, and in vivo validation [4,14,20,21,22]. Such integrated approaches may help identify peptide candidates for functional food and nutraceutical research; preventive or therapeutic applications require further evidence of efficacy and safety in physiologically relevant models. Representative plant protein sources, their major protein fractions, reported peptide-related activities, and key nutritional, technological, and safety-related limitations are summarized in Table 1.

**Table 1:** Representative plant protein sources, major protein fractions, and bioactive peptide-related functions.

Plant Source	Protein Content (% Dry Weight) *	Major Protein Fraction	Relevant Amino Acid/Protein Feature	Reported Peptide-Related Activity	Potential Allergenic Risk	Main Limitation/Challenge	Ref.
Soybean	~34–45	Glycinin, β-conglycinin	Lysine-rich legume globulins	ACE-inhibitory, lipid-regulating, antioxidant, and antidiabetic activities	Recognized major food allergen	Allergenicity; need for gastrointestinal stability and bioavailability validation	[14,19,20,21,22,32,33,34,71,72]
Pea	~15–22	Vicilin, legumin	Lysine-rich globulins	Antioxidant, metal-chelating, antihypertensive, and immunomodulatory activities	IgE-mediated allergy and cross-reactivity with other legumes reported	Bitterness and process-dependent peptide profiles after hydrolysis	[14,16,36,61,62]
Lentil	~20–26	Globulins, albumins	Relatively balanced legume protein profile	Antioxidant, ACE-inhibitory, enzyme-inhibitory, and anti-inflammatory activities	IgE-mediated allergy and legume cross-reactivity reported	Strong dependence on enzyme specificity and hydrolysis conditions	[14,16,35]
Chickpea	~18–25	Globulins, albumins	Lysine-rich protein matrix	Antioxidant and antidiabetic enzyme-inhibitory activities, including inhibition of DPP-IV, α-amylase, and α-glucosidase	IgE-mediated allergy and legume cross-reactivity reported	Antinutritional factors and potential allergenicity	[14,16,37,70,71,72]
Wheat	~10–15	Gliadins, glutenins	Proline- and glutamine-rich repetitive sequences	Bioactive and digestion-resistant peptides with potential immunomodulatory effects	Recognized major food allergen; gluten-related immunogenicity is a distinct additional concern	Gluten-related immunogenicity and coeliac-disease-associated responses	[4,29,44,73]
Rice	~7–15	Glutelins, rice bran proteins	Gluten-free cereal protein source; relatively low lysine content	Antioxidant, metal-chelating, and ACE-inhibitory activities	IgE-mediated allergy reported but relatively uncommon	Lower lysine content and limited confirmation of systemic bioavailability	[14,39,40]
Oat	~9–17	Globulins, avenins	Relatively balanced cereal amino acid profile	Antioxidant, pancreatic lipase-inhibitory, and α-amylase-inhibitory activities	Allergic reactions are relatively uncommon; avenin-related immune responses may occur in susceptible individuals	Processing- and hydrolysis-dependent activity	[14,41]
Quinoa	~13–15	Albumins, globulins	High-quality pseudocereal protein with a relatively balanced essential amino acid profile	Antioxidant, ACE-inhibitory, DPP-IV-inhibitory, antihypertensive, and immunomodulatory activities	Limited clinical evidence; occasional hypersensitivity reported	Limited in vivo and clinical validation	[15,25,45,46,47,48,49]
Amaranth	~14–17	Globulins, albumins	Lysine-rich pseudocereal protein	ACE-inhibitory, antithrombotic, antioxidant, and anti-inflammatory activities	Limited clinical evidence of allergenicity	Need for standardized hydrolysis and biological validation	[15,25,46,50,51,74]
Buckwheat	~12–15	Globulins, albumins	Relatively balanced amino acid profile	Cholesterol-lowering, antioxidant, and anti-inflammatory activities	Clinically relevant IgE-mediated allergenicity; severe reactions have been reported	Presence of clinically relevant allergenic storage proteins	[15,25,51,52,75,76]
Sunflower	~25–30	Helianthinin, albumins	Protein-rich oilseed processing by-product	Antioxidant, ACE-inhibitory, emulsifying, and oxidative-stabilizing properties	Seed allergy reported but relatively uncommon	Phenolic–protein interactions, colour, bitterness, and sensory limitations	[17,53,54,55]
Sesame	~20–28	11S globulin, 2S albumin	Protein containing sulfur-rich antioxidant peptide sequences	Antioxidant, ACE-inhibitory, and antihypertensive activities	Recognized major food allergen	Clinically relevant allergenicity, particularly associated with 2S albumin	[17,77,78,79]
Flaxseed	~20–25	Globulins, albumins	Arginine-rich protein capable of producing low-molecular-weight bioactive peptides	Antioxidant, ACE-inhibitory, and blood-pressure-lowering activities	IgE-mediated reactions are uncommon but documented	Mucilage, matrix complexity, and process-dependent peptide recovery	[17,60,80,81]
Chia	~16–24	Globulins, albumins	Arginine-rich protein fraction	Antioxidant, ACE-inhibitory, antihypertensive, and antidiabetic enzyme-inhibitory activities	Limited clinical evidence; hypersensitivity and potential cross-reactivity reported	Evidence remains predominantly in vitro; limited clinical validation	[17,82,83,84]
Hemp seed	~20–30	Edestin, albumin	Edestin-dominant, highly digestible oilseed protein	Antioxidant and ACE-inhibitory/antihypertensive activities	Limited clinical evidence; allergic reactions have been reported	Need for standardized extraction, hydrolysis, safety evaluation, and regulatory characterization	[17,85,86]

* Protein contents are approximate values expressed on a dry-weight basis and may vary according to cultivar, growing conditions, plant fraction, processing, and analytical method.

Allergenicity descriptions are qualitative and summarize currently reported clinical and immunological evidence; they should not be interpreted as standardized risk categories or as estimates of individual allergic risk.

## 4. Extraction, Hydrolysis, and Structural Characterization of Plant-Derived Peptides

Modern biotechnological workflows have substantially improved the extraction, production, purification, structural identification, and functional evaluation of bioactive peptides from plant matrices [6,7,8,9,10]. In contrast to chemically synthesized peptides, plant-derived peptides are obtained from complex biological materials in which proteins coexist with polysaccharides, lipids, phenolic compounds, pigments, antinutritional factors, minerals, and low-molecular-weight metabolites. Therefore, the efficient discovery of bioactive peptides requires a sequential and carefully optimized workflow involving raw material pretreatment, protein extraction, controlled hydrolysis, peptide fractionation, purification, sequence identification, bioinformatic prediction, and biological validation [6,8,10,87,88]. Each stage of this process directly affects peptide yield, sequence diversity, physicochemical properties, purity, reproducibility, and final biological activity [6,8,19,30,31]. The same plant source may generate different peptide profiles depending on extraction conditions, enzyme specificity, fermentation strategy, hydrolysis time, pH, temperature, pretreatment method, and purification approach. Consequently, methodological standardization is essential for comparing results across studies and for translating plant-derived peptide preparations into functional food, nutraceutical, or pharmaceutical applications [8,55,87,88].

### 4.1. Protein Extraction from Plant Matrices

Protein extraction is the first critical step in the generation of plant-derived bioactive peptides. The quality, purity, solubility, and structural integrity of the extracted protein fraction largely determine the subsequent hydrolysis profile and the diversity of peptides released. Because plant proteins differ markedly in solubility, localization, aggregation state, and interaction with non-protein matrix components, extraction conditions must be selected according to the botanical source and the dominant protein classes [14,15,16,17,89,90]. Legume and oilseed proteins are commonly extracted by alkaline solubilization followed by isoelectric precipitation. This approach is widely used because many storage globulins are soluble under alkaline conditions and precipitate near their isoelectric point. In contrast, cereal and pseudocereal proteins often require extraction systems adapted to their relative proportions of albumins, globulins, prolamins, and glutelins. For oil-rich seeds such as sunflower, sesame, hemp, flaxseed, pumpkin seed, and chia, defatting is usually performed before protein extraction to improve protein recovery, reduce lipid oxidation, and minimize lipid–protein interference during downstream processing [14,15,16,17]. The extraction process should maximize protein recovery while preserving functional groups and maintaining sufficient structural accessibility for subsequent enzymatic cleavage. Conventional alkaline extraction is simple, scalable, and efficient, but harsh pH conditions may induce protein denaturation, oxidation, deamidation, or aggregation. Therefore, milder and greener extraction strategies are increasingly being investigated to improve recovery while reducing solvent consumption and preserving protein functionality [89,90,91,92].

Aqueous and salt-based extraction methods are often preferred for albumins and globulins because they can maintain protein conformation under relatively mild and food-compatible conditions [90,91]. Enzyme-assisted extraction uses cell-wall-degrading enzymes such as cellulases, hemicellulases, and pectinases to disrupt polysaccharide barriers and increase protein accessibility [90,92]. Ultrasound-assisted extraction promotes acoustic cavitation, generating localized shear forces that disrupt cell walls and enhance mass transfer [89,90,91,92,93]. Microwave-assisted extraction accelerates heating of intracellular water and promotes matrix rupture, although excessive thermal exposure may induce protein modification [59,60,92]. High-pressure processing and pulsed electric fields are non-thermal or low-thermal approaches that alter cell membrane permeability and protein conformation, thereby facilitating protein release. Although these technologies can improve extraction efficiency, their parameters must be carefully optimized. Excessive ultrasound intensity may promote oxidation, high microwave power may induce thermal aggregation, and strong alkaline conditions may modify amino acid residues. Therefore, extraction efficiency should be evaluated together with protein integrity, solubility, digestibility, and peptide-generating potential rather than yield alone [59,60,89,90,91,92,93].

### 4.2. Enzymatic and Microbial Hydrolysis

Hydrolysis converts plant proteins into complex peptide mixtures with diverse molecular masses, sequences, charges, hydrophobicity, and biological properties. Enzymatic hydrolysis is the most widely used strategy because it enables relatively controlled peptide release under mild processing conditions. The selection of protease is a decisive factor because each enzyme exhibits characteristic substrate and cleavage-site preferences and consequently generates a distinct peptide profile [6,14,19,31,94].

Broad-specificity proteases such as alcalase are frequently used to achieve relatively high degrees of hydrolysis and release diverse short peptides. Trypsin preferentially cleaves peptide bonds at the C-terminal side of lysine and arginine residues, whereas chymotrypsin preferentially targets bonds adjacent to aromatic residues such as phenylalanine, tyrosine, and tryptophan [8,19,31,94]. Pepsin, pancreatin, papain, bromelain, Flavourzyme, and thermolysin are also widely applied depending on the plant protein substrate, desired peptide-size distribution, and target biological activity [6,14,94].

Enzyme specificity influences not only peptide length but also the exposure and terminal positioning of hydrophobic, aromatic, acidic, or basic amino acid residues [6,14,31,94]. Accordingly, proteases that release peptides containing hydrophobic or aromatic residues may favor the formation of antioxidant or ACE-inhibitory sequences, whereas preparations containing exopeptidase activity may reduce bitterness by progressively removing terminal hydrophobic residues [6,8,14,26,67,94].

The degree of hydrolysis is a key processing parameter representing the proportion of peptide bonds cleaved during enzymatic treatment. It influences peptide-size distribution, solubility, biological activity, sensory characteristics, digestibility, and techno-functional performance [6,55,58,65,94]. Moderate hydrolysis may improve solubility, emulsifying properties, digestibility, and selected biological activities, whereas excessive hydrolysis may produce free amino acids or very short peptides with reduced interfacial and techno-functional performance [6,55,58,65,94]. The degree of hydrolysis is commonly monitored using pH-stat, *o*-phthaldialdehyde, trinitrobenzenesulfonic acid, or free amino nitrogen assays [94,95]. Because these analytical methods measure different aspects of amino-group release and may not always produce directly comparable values, the selected method and calculation procedure should be clearly reported [95]. Microbial hydrolysis during fermentation provides a biologically complex and food-compatible alternative to hydrolysis using purified proteases. Lactic acid bacteria, *Bacillus* species, yeasts, and filamentous fungi produce endogenous proteases and peptidases that progressively degrade plant proteins and release peptides with potential biological activity [57,70,87]. The composition and concentration of the released peptides depend strongly on the microbial species and strain, proteolytic system, fermentation time, substrate composition, temperature, pH, water activity, and other processing conditions [87,96].

Fermentation may simultaneously reduce selected antinutritional factors, including phytates, tannins, and protease inhibitors; improve protein digestibility; modify sensory characteristics; and generate additional metabolites such as organic acids, exopolysaccharides, and transformed phenolic compounds. However, these effects are matrix-, strain-, and process-dependent and should therefore be confirmed experimentally rather than assumed for all fermented plant materials [57,70,87,96].

Microbial hydrolysis is generally more difficult to standardize than hydrolysis with purified enzymes because the resulting peptide profiles are influenced by microbial growth, strain-specific protease expression, substrate accessibility, fermentation conditions, and interactions with other matrix components [87,96]. Consequently, fermentation studies should clearly report the microorganism and strain, inoculum size, substrate composition, fermentation time, temperature, pH, and analytical methods used for peptide characterization.

Seed germination also promotes the activation of endogenous proteases and the mobilization of storage proteins. During germination, low-molecular-weight peptides and free amino acids may accumulate, while protein accessibility and digestibility can improve. This process is particularly relevant to cereals, legumes, and pseudocereals, in which controlled germination may improve nutritional quality and favor the release or formation of potentially bioactive peptide sequences [16,25,45,57,70].

The main proteolytic enzymes and microbial systems used for plant protein hydrolysis, together with their cleavage preferences and relevance to bioactive peptide generation, are summarized in Table 2.

Direct comparison among hydrolysis studies remains difficult because peptide profiles and reported activities are strongly influenced by protease specificity, enzyme-to-substrate ratio, degree of hydrolysis, pH, temperature, reaction time, and the subsequent fractionation strategy. Consequently, differences in biological activity reported for the same plant protein source may reflect methodological variation rather than intrinsic differences in the bioactive potential of the parent protein.

### 4.3. Fractionation and Purification of Peptide Mixtures

Crude plant protein hydrolysates contain complex mixtures of peptides, free amino acids, residual proteins, salts, carbohydrates, phenolic compounds, pigments, and other matrix-derived constituents. Fractionation is therefore necessary to enrich potentially bioactive peptides, reduce matrix interference, and improve analytical resolution [6,8,104,105]. A bioactivity-guided purification strategy is particularly useful, in which fractions exhibiting the highest activity are progressively separated, re-evaluated, and subjected to structural identification [104,106].

Ultrafiltration is commonly used as an initial fractionation step because it is relatively simple, scalable, and compatible with food-grade processing. Membranes with nominal molecular-weight cut-offs of 1, 3, 5, or 10 kDa enable the operational separation of peptide fractions according to approximate molecular size. However, membrane separation is not based exclusively on molecular mass, because membrane material, pore-size distribution, peptide charge, hydrophobicity, concentration polarization, and peptide–membrane interactions can also influence retention and permeation [105].

Low-molecular-weight fractions, particularly those below 3 kDa or 1 kDa, are frequently prioritized because they often exhibit strong antioxidant, ACE-inhibitory, or antidiabetic activities and may have greater potential for gastrointestinal stability and intestinal transport [20,21,22,61,62,63,64,105,107]. Nevertheless, low molecular weight should not be considered an independent predictor of biological activity. Peptide sequence, amino acid composition, terminal residues, hydrophobicity, net charge, amphiphilicity, conformation, and resistance to enzymatic degradation are also critical determinants [14,26,61,104,106].

Chromatographic purification provides higher resolution than membrane fractionation and enables the enrichment or isolation of individual peptide populations and, in some cases, purified peptide sequences. Reversed-phase high-performance liquid chromatography separates peptides primarily according to differences in hydrophobic interactions with the stationary phase and is widely used as a final or near-final purification step before mass-spectrometric identification [8,104,105,106,107,108].

Ion-exchange chromatography separates peptides according to differences in net charge and is useful for resolving acidic, neutral, and basic peptide populations. Size-exclusion chromatography separates peptides according to hydrodynamic size and is frequently used for molecular-size profiling or intermediate purification, although its ability to resolve peptides of similar size may be limited [104,106,108]. Hydrophilic interaction liquid chromatography can complement reversed-phase separation for highly polar or hydrophilic peptides that exhibit weak retention on conventional reversed-phase columns [104,105].

In practice, no single separation technique is sufficient to resolve all components of a complex plant protein hydrolysate. Multiple orthogonal methods are therefore commonly combined to exploit differences in molecular size, charge, hydrophobicity, and polarity. For example, a hydrolysate may initially be fractionated by ultrafiltration, followed by ion-exchange or size-exclusion chromatography, reversed-phase HPLC, and mass-spectrometric identification of peptides present in the most active fractions [104,108].

This stepwise, bioactivity-guided approach increases the probability of identifying peptides associated with a particular biological effect. However, biological activity should be reassessed after every purification stage because activity may arise from a single peptide, additive or synergistic interactions among several peptides, or contributions from non-peptide matrix components [104,105]. Comparison of activity should also account for peptide concentration, recovery yield, purification fold, and the analytical method used, rather than relying only on the apparent activity of the final fraction [105,106].

### 4.4. Mass Spectrometric Identification and Structural Characterization

High-resolution mass spectrometry is central to modern plant peptidomics because it enables the sensitive detection, molecular-mass determination, structural characterization, and sequence identification of peptides in complex biological matrices. Liquid chromatography coupled with tandem mass spectrometry enables peptide separation, ionization, detection, fragmentation, and sequence assignment within a single analytical workflow [8,104,108,109].

Electrospray ionization is commonly used for online LC–MS/MS analysis because it can directly couple liquid chromatographic separation with mass-spectrometric detection. Matrix-assisted laser desorption/ionization time-of-flight mass spectrometry is particularly useful for rapid peptide-mass profiling and the analysis of relatively simple peptide fractions. High-resolution instruments, including quadrupole time-of-flight and Orbitrap mass spectrometers, provide accurate mass measurements, high sensitivity, broad dynamic ranges, and improved confidence in molecular-formula and peptide-sequence assignments [104,108,109].

Tandem mass spectrometry generates peptide-sequence information through the controlled fragmentation of selected precursor ions. Collision-induced dissociation and higher-energy collisional dissociation primarily promote peptide-backbone cleavage and commonly generate complementary b- and y-type fragment ions used for sequence assignment [109,110]. Electron-transfer dissociation produces complementary fragmentation patterns, particularly *c*- and *z*-type ions, and can preserve labile post-translational modifications that may be partially or completely lost during collision-based fragmentation. Combining complementary fragmentation methods can therefore increase sequence coverage and improve the localization of structurally labile modifications [110].

Confidence in peptide identification is strengthened when fragment-ion matching is evaluated together with accurate precursor mass, isotope distribution, chromatographic retention behavior, charge state, and, when available, ion-mobility information [10,109,111,112,113]. However, accurate precursor mass alone is insufficient for unambiguous sequence identification because different peptide sequences, isomeric residues, post-translational modifications, or co-eluting compounds may produce identical or closely related mass-to-charge ratios [104,109,112]. For clarity, peptide identifications should be reported according to the level of analytical confidence. In this review, sequences inferred primarily from precursor mass, database matching, or incomplete fragmentation evidence are considered putative identifications. Higher-confidence sequence assignments require consistent MS/MS fragment-ion evidence together with appropriate peptide-spectrum-match validation and false-discovery-rate control. Confidence can be further strengthened by manual inspection of diagnostic fragment ions, targeted MS/MS analysis, chromatographic retention-time matching, and, where feasible, comparison with an authentic or synthetic peptide standard [10,103,108,110,111]. Accordingly, the terms “identified” or “experimentally confirmed” should be reserved for sequences supported by sufficient orthogonal analytical evidence, whereas tentative assignments should be explicitly described as putative.

Because many plant species remain incompletely represented in genomic and proteomic databases, de novo peptide sequencing is often required for the characterization of previously unreported plant-derived peptides. De novo sequencing reconstructs candidate amino acid sequences directly from MS/MS fragment-ion spectra without complete dependence on a reference protein database [10,111]. Its reliability nevertheless depends on spectral quality, fragmentation completeness, precursor-mass accuracy, peptide length, charge state, and the ability of the algorithm to distinguish isobaric or isomeric residues [111].

When high-quality genomic, transcriptomic, or protein-sequence databases are available, database-assisted searching can improve peptide-identification efficiency and provide information about the possible parent proteins from which the peptides originated. Hybrid approaches combining database searching with de novo sequencing are particularly valuable for non-model plant species, incomplete protein databases, unexpected enzymatic cleavage products, and peptides containing previously unreported modifications [10,104,111].

In both database-dependent and de novo workflows, rigorous control of peptide-spectrum matches and false discovery rates is essential to prevent the overinterpretation of complex peptidomic datasets [109,112]. Target–decoy strategies are widely used to estimate false discovery rates; however, the applied threshold, database composition, search space, peptide-level filtering criteria, and validation procedure should be clearly reported [112]. Where possible, high-confidence peptide assignments should additionally be supported by manual inspection of key fragment ions, comparison with synthetic standards, retention-time matching, or targeted MS/MS analysis.

Ion mobility spectrometry provides an additional gas-phase separation dimension based on ion mobility, which is influenced by molecular size, shape, charge, and collision cross section. When coupled with high-resolution mass spectrometry, ion mobility can separate co-eluting ions and distinguish some isomeric or conformationally different peptides that exhibit identical or closely related mass-to-charge ratios [113].

This additional separation dimension is particularly valuable for complex plant protein hydrolysates containing modified peptides, structural isomers, multiple charge states, and co-eluting matrix components. Nevertheless, ion-mobility separation alone does not establish peptide identity; reliable structural assignment requires its integration with accurate mass, retention time, fragmentation spectra, database or de novo sequence analysis, and, when necessary, comparison with authentic peptide standards [104,109,111,113].

### 4.5. Complementary Structural and Functional Analyses

Although mass spectrometry provides peptide sequence and molecular-mass information, complementary structural techniques are required to characterize peptide conformation, aggregation state, molecular interactions, and structure–activity relationships [104,109,114]. Circular dichroism spectroscopy is widely used to estimate peptide secondary-structure elements, including α-helical, β-sheet, β-turn, and disordered conformations in solution [114]. However, CD spectra should generally be interpreted as estimates of the average conformational population rather than as definitive three-dimensional structural assignments.

Fourier-transform infrared spectroscopy provides complementary information on peptide backbone conformation, particularly through analysis of the amide I and amide II spectral regions [114]. The amide I region is especially sensitive to peptide secondary structure because it is dominated by backbone carbonyl stretching vibrations, although band overlap, hydration, aggregation, and sample-preparation conditions may complicate spectral interpretation [114].

Nuclear magnetic resonance spectroscopy can provide residue-level information on peptide three-dimensional structure, conformational dynamics, intramolecular interactions, and binding to molecular partners. Depending on peptide size and solubility, one- and multidimensional NMR methods can be applied to determine chemical shifts, coupling patterns, nuclear Overhauser effects, and conformational restraints [114]. Fluorescence spectroscopy is useful for evaluating peptide interactions with proteins, lipid membranes, nucleic acids, and metal ions through changes in fluorescence intensity, emission wavelength, anisotropy, or fluorophore accessibility. Nevertheless, fluorescence quenching data should be interpreted carefully because inner-filter effects, peptide aggregation, and changes in the local environment may influence the observed signal.

Functional validation is essential after peptide identification because structural assignment alone does not demonstrate biological activity [8,104]. Antioxidant potential may initially be evaluated using radical-scavenging assays, reducing-power measurements, metal-chelation tests, or lipid-peroxidation inhibition models [115]. Because individual chemical assays reflect different antioxidant mechanisms and are strongly influenced by reaction medium and assay conditions, antioxidant activity should preferably be assessed using more than one complementary method [115]. Cellular oxidative-stress models provide stronger biological relevance by evaluating whether peptides can reduce intracellular reactive oxygen species, oxidative damage, or stress-responsive signaling under physiologically relevant conditions.

Antihypertensive potential is frequently evaluated using angiotensin-converting enzyme inhibition assays, followed, where appropriate, by enzyme-kinetic studies, simulated gastrointestinal digestion, cellular models, and in vivo blood-pressure assessment [14,19,32,33,34]. Antidiabetic activity may be investigated through inhibition of DPP-IV, α-amylase, or α-glucosidase; however, comparison among studies requires careful reporting of enzyme source, substrate, peptide concentration, positive control, incubation conditions, and the method used to calculate inhibitory activity [116].

Antimicrobial activity is commonly evaluated by broth microdilution-based minimum inhibitory concentration determination, growth-kinetic analysis, membrane-permeabilization assays, and complementary studies of microbial membrane integrity or intracellular targets [117]. Assay conditions such as medium composition, salt concentration, inoculum density, peptide adsorption, plate material, and the presence of serum components may substantially affect the apparent activity of antimicrobial peptides. Therefore, standardized and fully reported susceptibility-testing conditions are essential for meaningful comparison among peptide studies [117].

Anti-inflammatory and immunomodulatory effects may be assessed in cell-based models by measuring cytokine expression or secretion, nitric oxide production, NF-κB or related signaling pathways, oxidative-stress markers, and macrophage activation or polarization. These assays should include appropriate controls for cytotoxicity because an apparent reduction in inflammatory mediators may result from loss of cell viability rather than a specific anti-inflammatory effect.

Chemical, enzyme-based, and simplified cell-free assays alone are insufficient to establish physiological relevance. Promising peptides should additionally be evaluated for cytotoxicity, gastrointestinal stability, resistance to intestinal and plasma peptidases, intestinal epithelial transport, allergenic potential, and activity in appropriate cellular or in vivo models [20,21,22,61,62,63,64,118]. Gastrointestinal digestion, mucus, epithelial barriers, peptide transport mechanisms, and interactions with other food components can substantially influence whether an orally administered peptide reaches the circulation or its molecular target in an active form [118].

This validation sequence is particularly important because peptides displaying strong activity in chemical or enzyme-based assays may be degraded during gastrointestinal digestion, undergo structural modification, exhibit limited epithelial permeability, or fail to reach target tissues at biologically effective concentrations [20,21,22,61,62,63,64,119]. Accordingly, biological claims should be proportional to the level of experimental validation: activity observed exclusively in a chemical assay should not be interpreted as evidence of in vivo efficacy.

### 4.6. Bioinformatics and Peptidomic Data Processing

Bioinformatics has become an indispensable component of plant peptide discovery because modern peptidomic workflows generate large and multidimensional datasets that cannot be interpreted reliably by manual analysis alone [10,104,119]. High-resolution LC–MS/MS and ion-mobility datasets contain complex information on precursor ions, isotope patterns, retention times, charge states, fragment ions, and gas-phase mobility, requiring advanced computational processing before confident peptide identification and quantitative comparison can be achieved [10,109,113,120].

Peak detection, spectral deconvolution, isotope clustering, retention-time alignment, feature matching, and fragment-ion annotation are essential steps in the conversion of raw mass-spectrometric data into interpretable peptide features. Database-search tools such as Mascot, SEQUEST, PEAKS, MaxQuant, and related proteomic platforms match experimental MS/MS spectra against theoretical spectra generated from reference protein databases [10,109,111,120]. The reliability of these searches depends on database completeness, precursor- and fragment-mass tolerances, enzyme specificity, permitted missed cleavages, variable and fixed modifications, and the scoring and filtering criteria applied.

Target–decoy database strategies are widely used to estimate false discovery rates at the peptide-spectrum-match, peptide, or protein level [112,120]. An FDR threshold of approximately 1% is frequently applied for high-confidence identifications, but this value should not be interpreted as a universal guarantee of correctness because FDR performance depends on the search space, database construction, scoring method, dataset complexity, and the level at which error rates are calculated [112]. Consequently, studies should explicitly report the target–decoy strategy, FDR level, threshold, database version, search parameters, and post-search validation criteria.

In non-model plants with incomplete or poorly annotated protein databases, de novo sequencing and hybrid approaches combining database searching with de novo-derived sequence tags are particularly valuable [10,104,111,119]. These methods can recover candidate sequences that would otherwise remain unidentified because of missing reference proteins, unexpected cleavage products, sequence polymorphisms, or post-translational modifications. Nevertheless, de novo assignments should be interpreted cautiously and, where possible, confirmed through manual fragment-spectrum inspection, targeted MS/MS analysis, comparison with genomic or transcriptomic data, or synthetic peptide standards [111].

Label-free quantification based on chromatographic peptide peak areas, ion intensities, or spectral counts enables comparison of peptide abundance among different extraction, hydrolysis, germination, or fermentation conditions [120]. Intensity-based and spectral-counting approaches are not analytically equivalent, and intensity-based measurements generally provide broader quantitative resolution, whereas spectral counting may be less reliable for low-abundance peptides. Robust label-free analysis requires appropriate biological and technical replication, retention-time alignment, feature normalization, missing-value assessment, quality control, and statistical correction for multiple comparisons [120].

Multivariate statistical methods, including principal component analysis, hierarchical clustering, partial least-squares regression, and correlation analysis, can be used to explore relationships between peptide profiles and measured biological activities [10,119,120]. These approaches may identify peptide features associated with antioxidant, antihypertensive, antimicrobial, or antidiabetic effects. However, statistical association does not by itself establish that an individual peptide is responsible for the observed activity, particularly when active fractions contain multiple peptides or residual non-peptide compounds. Candidate markers therefore require targeted purification, synthesis, or orthogonal experimental validation.

In silico tools further support peptide prioritization by predicting physicochemical properties, solubility, hydrophobicity, net charge, amphiphilicity, toxicity, allergenicity, protease susceptibility, and potential biological activity [10,119,121]. The reliability of these predictions depends strongly on the quality, diversity, class balance, and experimental consistency of the datasets used to train and validate the predictive models [121]. Predictions may be less reliable for unusual amino acids, modified peptides, underrepresented plant sequences, or peptide classes that differ substantially from the training data.

Molecular docking can generate hypotheses regarding possible peptide-binding modes and interactions with target enzymes or receptors, while molecular-dynamics simulations can evaluate the temporal stability and conformational behavior of proposed complexes. Machine-learning and protein-language-model approaches can further rank candidate peptides for experimental validation and have already been applied to the prediction of specific activities, including ACE inhibition [121,122]. Nevertheless, docking scores, predicted binding poses, and machine-learning classifications should not be considered direct evidence of biological activity or therapeutic efficacy.

Computational predictions should therefore be regarded as hypothesis-generating and prioritization tools rather than substitutes for biochemical and biological testing [10,119,121,122]. The strongest discovery pipelines integrate computational prediction with high-confidence MS/MS identification, targeted synthesis or purification, standardized activity assays, stability and transport studies, and validation in relevant cellular or in vivo models.

### 4.7. Proposed Workflow for Plant-Derived Bioactive Peptide Discovery

A systematic workflow for the discovery of plant-derived bioactive peptides should integrate biochemical, analytical, computational, and biological approaches [8,10,104,119]. The process typically begins with the selection and pretreatment of plant biomass, including cleaning, drying, milling, and, when necessary, defatting. These preliminary operations should be standardized because the botanical source, cultivar, tissue type, storage conditions, particle size, moisture content, and lipid content may influence protein recovery and the composition of the resulting peptide fractions [17,89,90,91,92,93].

Protein extraction is then carried out using conventional alkaline or salt-based methods, enzyme-assisted extraction, or emerging green technologies, depending on the characteristics of the plant matrix and the intended downstream application [59,60,89,90,91,92,93]. The selected extraction conditions should maximize protein recovery while limiting oxidation, aggregation, denaturation, and irreversible modification of amino acid residues. The principal advantages and limitations of these extraction strategies are compared in Table 3.

The extracted proteins are subsequently subjected to controlled peptide-release strategies, including enzymatic hydrolysis, simulated gastrointestinal digestion, microbial fermentation, or germination-induced proteolysis [14,16,19,31,87]. Protease specificity, enzyme-to-substrate ratio, degree of hydrolysis, reaction time, pH, and temperature should be optimized because these variables determine peptide-size distribution, sequence composition, physicochemical properties, sensory characteristics, and biological activity [6,19,58,65,94,95].

The resulting hydrolysates are fractionated by ultrafiltration and chromatographic techniques, and the obtained fractions are screened for targeted biological activities [8,104,105,106,107,108]. Bioactivity-guided purification is particularly useful, provided that biological activity is reassessed after each fractionation step and interpreted together with peptide recovery, concentration, purification fold, and possible synergistic interactions among components [104,105,106,107,108].

Active fractions are then analyzed using advanced mass-spectrometric platforms, including LC–MS/MS, MALDI-TOF MS, Q-TOF or Orbitrap systems, and, where appropriate, ion-mobility mass spectrometry, to identify peptide sequences and structural features potentially associated with bioactivity [104,109,113]. High-confidence identification should combine accurate precursor mass, fragment-ion matching, retention-time information, isotope patterns, and rigorous false-discovery-rate control. In non-model plants with incomplete sequence databases, de novo sequencing and hybrid database-assisted approaches are particularly important [10,104,111,112]. As summarized in Table 3, these downstream fractionation, purification, and mass-spectrometric approaches provide complementary advantages but also differ in resolution, throughput, cost, sample requirements, and structural information.

In parallel, bioinformatic and computational tools can be employed to predict peptide physicochemical properties, biological activity, stability, toxicity, allergenicity, protease susceptibility, and potential interactions with molecular targets [10,119,120,121,122]. Molecular docking, molecular-dynamics simulations, machine-learning models, and protein-language-model approaches may assist in prioritizing candidate peptides, but these predictions should be regarded as hypothesis-generating tools rather than direct evidence of biological efficacy [10,119,121,122].

Selected high-ranking peptide candidates may then be chemically synthesized or purified in larger amounts and subjected to orthogonal experimental validation. This stage should include confirmation of identity and purity, concentration-dependent biochemical assays, appropriate positive and negative controls, cytotoxicity assessment, and validation in relevant cellular and, where justified, in vivo models [8,104,114,117]. Comparison of purified or synthesized peptides with the original active fraction can also help determine whether the observed effect is attributable to a single peptide or to additive or synergistic interactions among several components.

Finally, assessment of gastrointestinal and plasma stability, intestinal transport, bioavailability, allergenicity, dose–response relationships, and safety is essential for determining the suitability of peptide candidates for application in functional foods, nutraceuticals, or therapeutic formulations [61,62,63,64,118]. Strong activity in a chemical or enzyme-based assay alone should not be interpreted as evidence of physiological or therapeutic efficacy.

**Figure 1:**
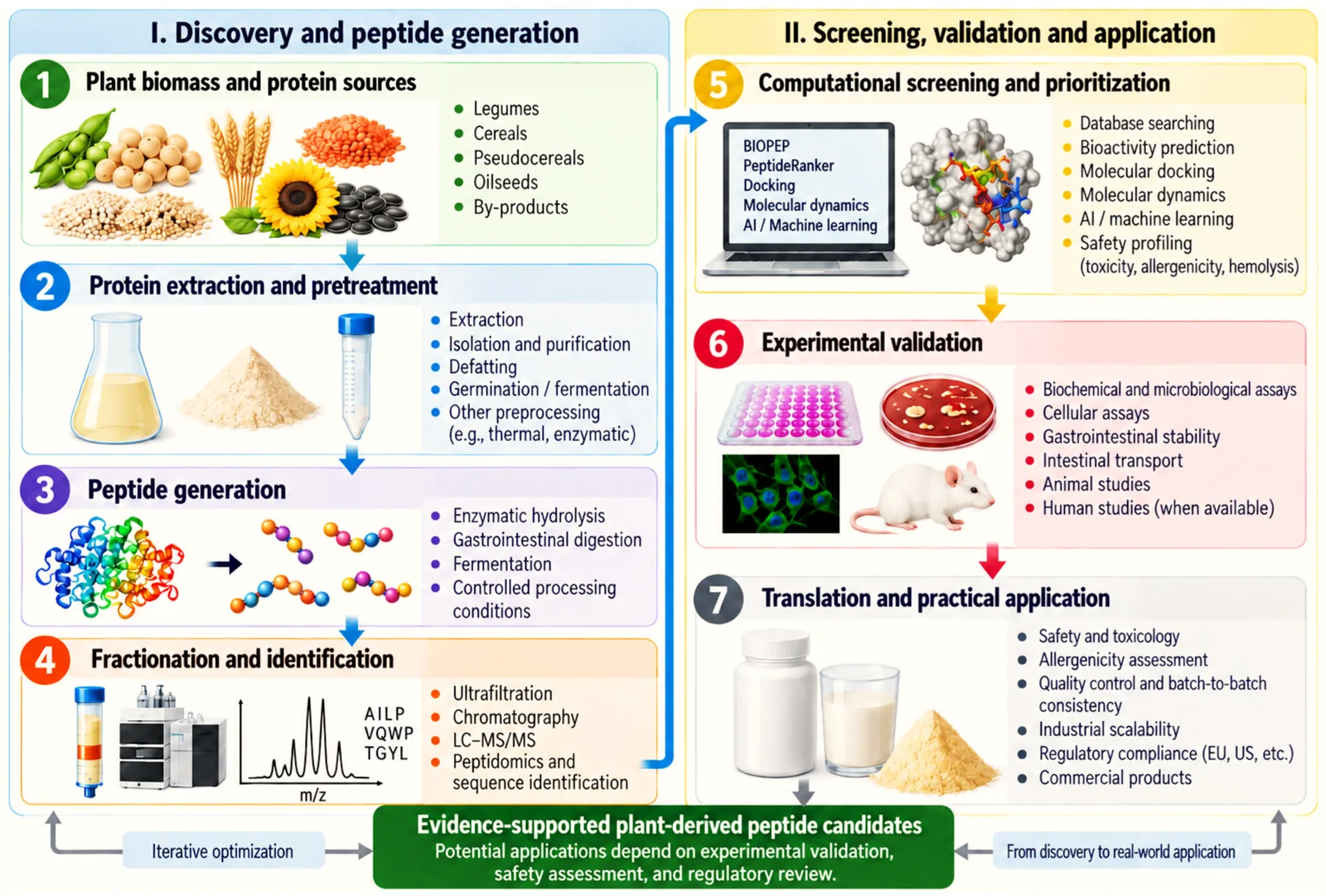
Conceptual workflow for the discovery and validation of plant-derived bioactive peptides.

As summarized in Figure 1, this integrated workflow supports the transition from crude plant protein hydrolysates to structurally characterized and biologically validated peptide candidates. It also improves reproducibility, facilitates comparison among studies, reduces the risk of false-positive candidate selection, and enables the rational prioritization of promising peptides for translational development [8,10,104,119,120].

The scheme summarizes the major stages involved in transforming protein-rich plant materials into structurally characterized and biologically validated peptide candidates. The workflow includes plant-source selection and standardized pretreatment, protein extraction, controlled peptide release through hydrolytic or biotechnological processing, peptide fractionation and structural identification, functional and computational screening, orthogonal experimental validation, and final assessment of stability, bioavailability, safety, and application potential.

The main techniques used for protein extraction, peptide generation, fractionation, purification, and structural characterization, together with their key advantages and limitations, are summarized in Table 3.

## 5. Biological Activities and Computational Approaches for Predicting Plant Peptide Function

Plant-derived peptides exhibit a broad spectrum of biological activities mediated through interactions with enzymes, receptors, biological membranes, transporters, transcriptional regulators, and intracellular signaling pathways [1,2,3,4,14,18,19]. Once released from their parent proteins during gastrointestinal digestion, controlled enzymatic hydrolysis, microbial fermentation, germination, or food processing, these peptides may act locally within the gastrointestinal tract or, if they remain stable and are absorbed, exert systemic effects in target tissues [1,61,62,63,64,118].

Their biological functions depend on interconnected structural and physicochemical features, including amino acid sequence, peptide length, molecular weight, hydrophobicity, net charge, amphipathicity, secondary structure, conformational flexibility, terminal residues, and resistance to proteolytic degradation [1,2,3,14,18,20]. Consequently, peptides with similar amino acid compositions may exhibit markedly different activities when their residue order, terminal positioning, conformation, or charge distribution differs.

The functional diversity of plant-derived peptides is closely related to the chemical properties and spatial arrangement of their amino acid residues [1,2,3,18,26,28]. Hydrophobic residues, including leucine, isoleucine, valine, alanine, phenylalanine, and proline, can promote interactions with lipid membranes, hydrophobic regions of proteins, and nonpolar pockets within enzymes or receptors. However, excessive hydrophobicity may also decrease aqueous solubility, promote aggregation, or increase nonspecific interactions [1,3,18,26].

Aromatic residues, particularly phenylalanine, tyrosine, and tryptophan, may contribute to antioxidant activity through electron or hydrogen-atom donation and stabilization of radical intermediates. These residues can also participate in hydrophobic and π-related interactions with molecular targets [1,2,3,18,26,115]. Nevertheless, their contribution depends on residue accessibility, sequence position, neighboring amino acids, peptide conformation, and the assay environment.

Positively charged residues, such as lysine and arginine, can enhance electrostatic interactions with negatively charged microbial membranes, nucleic acids, or acidic regions of proteins. Histidine may also contribute to electrostatic interactions, metal coordination, proton transfer, and antioxidant activity, although its behavior is strongly influenced by pH and the local peptide environment [1,2,3,26,28]. The combination of positive charge and appropriate hydrophobicity is particularly relevant to membrane-active antimicrobial peptides, but neither property alone is sufficient to predict antimicrobial potency or selectivity [1,3,28,117].

Sulfur-containing residues, particularly cysteine and methionine, can participate in redox-related mechanisms and may contribute to radical scavenging or structural stabilization. Cysteine may additionally form disulfide bonds that constrain peptide conformation and influence stability, whereas methionine can undergo reversible oxidation [1,3,14,26,28]. Acidic residues, including aspartic acid and glutamic acid, may contribute to metal-ion coordination, electrostatic recognition, and interactions with enzymes or receptors. However, metal chelation generally depends on the combined contribution of several coordinating groups and their spatial arrangement rather than on the presence of an individual acidic residue alone [1,3,26,115].

Therefore, peptide activity is determined not only by botanical origin or bulk amino acid composition, but also by sequence order, residue position, terminal composition, three-dimensional conformation, charge distribution, amphipathicity, stability, target accessibility, concentration, and biological context [1,2,3,18,26,61,62]. Structure–activity relationships should consequently be evaluated using purified or synthesized peptides and supported, where possible, by biochemical, biophysical, cellular, computational, and in vivo validation. Differences among reported activity values should be interpreted cautiously, as they may arise from variation in peptide purity, assay format, enzyme or substrate source, concentration units, incubation conditions, and biological model. Therefore, numerical activities obtained in different studies should not be considered directly comparable unless the experimental conditions are sufficiently similar.

To distinguish general hydrolysate-level observations from sequence-specific experimental evidence, representative plant-derived peptides that have undergone direct experimental validation are summarized in Table 4. Priority is given to studies in which individual peptide sequences were analytically identified and subsequently synthesized, purified, or otherwise independently tested for biological activity. The selected examples illustrate different levels of evidence, ranging from biochemical and cellular validation to gastrointestinal stability, intestinal transport, animal studies, and human bioavailability.

### 5.1. Antioxidant Activity

Oxidative stress is characterized by an imbalance between the production of reactive oxygen species (ROS) and reactive nitrogen species (RNS) and the capacity of cellular antioxidant systems to neutralize or regulate these reactive species. Plant-derived antioxidant peptides may counteract oxidative stress through several complementary mechanisms, including direct radical scavenging, electron or hydrogen-atom donation, transition-metal chelation, inhibition of lipid peroxidation, and modulation of cellular redox-regulatory pathways [1,2,3,117,129,130,131].

Beyond direct chemical antioxidant activity, selected peptides may influence endogenous antioxidant defense systems by modulating redox-sensitive signaling pathways, particularly the nuclear factor erythroid 2-related factor 2 (Nrf2) pathway. Activation and nuclear translocation of Nrf2 can enhance the transcription of antioxidant and cytoprotective genes, including those encoding heme oxygenase-1 (HO-1), superoxide dismutase (SOD), catalase (CAT), glutathione peroxidase (GPx), and enzymes involved in glutathione metabolism [130,132]. However, Nrf2/HO-1 activation should be demonstrated experimentally for individual peptides and should not be assumed solely from radical-scavenging activity measured in chemical assays.

The antioxidant potential of plant-derived peptides is strongly influenced by amino acid composition, sequence order, residue position, peptide length, terminal residues, conformation, and accessibility of reactive side chains. Peptides containing tyrosine, tryptophan, phenylalanine, methionine, cysteine, histidine, or proline frequently exhibit antioxidant or lipid-protective potential, although the contribution of each residue depends on its position within the sequence and the surrounding molecular environment [1,3,26,27,131].

Aromatic residues, particularly tyrosine and tryptophan, may stabilize radical intermediates through resonance and participate in electron- or hydrogen-atom-transfer reactions. Sulfur-containing residues, especially cysteine and methionine, can participate in oxidation–reduction reactions, whereas histidine may contribute to proton transfer and transition-metal coordination. Hydrophobic residues may enhance peptide interactions with lipid phases and facilitate protection against lipid oxidation. Nevertheless, excessive hydrophobicity can reduce aqueous solubility or promote aggregation; therefore, the presence of a particular residue alone does not reliably predict antioxidant activity [1,3,26,27,131].

Antioxidant activity is commonly screened using chemical assays such as DPPH, ABTS, FRAP, ORAC, reducing-power, hydroxyl-radical-scavenging, superoxide-radical-scavenging, lipid-peroxidation-inhibition, and metal-chelating assays. These methods measure different reaction mechanisms and are not analytically interchangeable. DPPH, ABTS, and FRAP assays predominantly reflect electron-transfer-related behavior under defined chemical conditions, whereas ORAC is principally based on hydrogen-atom-transfer reactions. Assay outcomes may also be influenced by solvent composition, pH, reaction time, peptide concentration, radical source, and the selected reference standard [115,131].

A sequence-resolved example is the tetrapeptide EAAY (Glu-Ala-Ala-Tyr) identified from *Paeonia ostii* ‘Feng Dan’ seed meal by nano-LC–MS/MS. At 0.5 mg/mL, EAAY showed 98.5 ± 1.1% ABTS radical-scavenging activity and 61.9 ± 1.3% hydroxyl-radical-scavenging activity, while no obvious cytotoxicity was observed toward L929 fibroblast cells [18]. This example illustrates the importance of validating antioxidant activity at the level of an identified peptide rather than attributing activity solely to the parent hydrolysate [18] (Table 4).

Because no single chemical assay represents all antioxidant mechanisms, antioxidant peptide preparations should preferably be evaluated using multiple complementary assays and reported with clearly defined experimental conditions [115,131].

However, even strong activity in several cell-free assays does not establish physiological antioxidant efficacy because these systems do not reproduce cellular uptake, metabolism, peptide degradation, subcellular localization, or interactions with biological macromolecules.

Cell-based models are therefore increasingly used to evaluate peptide protection against intracellular ROS accumulation, mitochondrial dysfunction, lipid peroxidation, protein oxidation, DNA damage, and disruption of endogenous antioxidant defenses [129,130]. Such studies may also assess Nrf2 nuclear translocation, HO-1 expression, glutathione status, antioxidant-enzyme activity, mitochondrial membrane potential, and oxidative-stress-responsive signaling pathways.

Appropriate controls are essential when interpreting cell-based antioxidant activity. Cytotoxicity should be evaluated independently because an apparent reduction in ROS may result from reduced cell viability or metabolic activity. In addition, peptide stability in the culture medium, cellular uptake, dose–response behavior, and comparison with established antioxidant controls should be considered. These models help distinguish peptides that act primarily as chemical radical scavengers from those capable of protecting cells or modulating cellular redox homeostasis. Nevertheless, confirmation in gastrointestinal stability, intestinal transport, animal, or human studies remains necessary before physiological or health-related antioxidant claims can be made [20,22,64,130,131].

### 5.2. Antimicrobial Activity

Plant-derived antimicrobial peptides represent a promising group of natural compounds with potential applications in food preservation, crop protection, biotechnology, and anti-infective research [1,2,28,119,133]. These peptides have been reported to inhibit the growth of Gram-positive and Gram-negative bacteria, yeasts, and filamentous fungi, whereas antiviral effects remain less extensively characterized and require further mechanistic and biological validation [1,2,3,133].

Their antimicrobial activity is commonly associated with a combination of cationic charge, amphipathic structure, hydrophobicity, conformational flexibility, and the ability to interact selectively with microbial cell envelopes [1,3,28,132,133]. However, no single physicochemical property is sufficient to predict antimicrobial potency or selectivity. Peptide length, sequence order, charge distribution, secondary structure, aggregation tendency, salt sensitivity, and stability in biological media also strongly influence activity.

Positively charged peptides can interact electrostatically with negatively charged microbial surface components, including anionic phospholipids, lipopolysaccharides in Gram-negative bacteria, and lipoteichoic and teichoic acids in Gram-positive bacteria [28,132,133]. Following surface binding, peptides may insert into or destabilize the membrane, increase permeability, induce membrane depolarization, promote leakage of intracellular constituents, or cause extensive bilayer disruption.

Several idealized biophysical models have been proposed to describe peptide-induced membrane permeabilization [132,133,134]. In the barrel-stave model, peptides insert approximately perpendicular to the lipid bilayer and oligomerize to form transmembrane channel-like structures. In the toroidal-pore model, peptides induce continuous bending of the lipid monolayers, producing pores lined by both peptide molecules and lipid head groups. In the carpet model, peptides accumulate largely parallel to the membrane surface and, after reaching a critical surface concentration, disrupt the bilayer in a detergent-like manner [133,134].

These models should be regarded as conceptual frameworks rather than universally distinct mechanisms. Individual antimicrobial peptides may exhibit mixed, transient, concentration-dependent, or membrane-composition-dependent behavior, and stable barrel-stave or toroidal pores have been structurally demonstrated for only a limited number of peptides [133,134]. Consequently, assignment of a particular membrane-disruption model should ideally be supported by complementary biophysical methods rather than inferred solely from antimicrobial activity.

Beyond membrane disruption, some plant-derived antimicrobial peptides may cross microbial membranes and act on intracellular targets, including DNA, RNA, ribosomes, protein-folding machinery, metabolic enzymes, cell-division proteins, and cell-wall-biosynthetic pathways [132,135]. Other peptides may combine membrane permeabilization with intracellular inhibition, producing a multistep or multitarget mode of action.

Such mechanistic diversity may make the development of resistance more complex than for some single-target antibiotics; however, antimicrobial peptides should not be considered inherently resistance-proof. Microorganisms may reduce peptide susceptibility through surface-charge modification, membrane remodeling, extracellular or intracellular proteases, efflux systems, biofilm formation, and stress-response pathways. Therefore, resistance development should be evaluated experimentally through serial-passage, mutant-selection, or related adaptation studies.

Antimicrobial activity is commonly evaluated using agar diffusion assays, broth microdilution for minimum inhibitory concentration determination, time–kill kinetics, membrane-depolarization and permeability assays, leakage measurements, flow cytometry, and microscopy-based approaches [117]. Agar diffusion is useful for preliminary screening but may underestimate the activity of peptides that diffuse poorly or bind to agar components. Broth microdilution is generally more appropriate for quantitative MIC determination, although medium composition, ionic strength, inoculum density, peptide adsorption to plastic surfaces, incubation conditions, and serum components may substantially influence the measured activity [117,136].

A representative sequence-resolved example is MOp3 (MCNDCGA)**,** an antimicrobial peptide identified by LC–MS/MS from *Moringa oleifera* seed protein hydrolysates. MOp3 showed inhibitory activity against *Staphylococcus aureus* with a reported MIC of 2 mg/mL and exhibited negligible hemolytic activity at 2 × MIC. Microscopic analysis indicated irreversible damage to the bacterial cell membrane, while additional molecular studies suggested interactions with dihydrofolate reductase and DNA gyrase. This example illustrates the importance of reporting antimicrobial peptide sequence, quantitative MIC values, selectivity, and mechanistic evidence together when evaluating antimicrobial potential [137].

Because membrane-active peptides may also interact with mammalian cell membranes, antimicrobial potency should be interpreted together with selectivity and safety. Hemolysis assays, mammalian-cell cytotoxicity testing, serum and protease stability, and calculation of a therapeutic or selectivity index are therefore essential before practical application [117,136]. Hemolysis measurements should also be standardized because erythrocyte source and concentration, incubation time, peptide concentration, buffer composition, and calculation method can markedly affect the reported result [136].

Accordingly, promising plant-derived antimicrobial peptides should be evaluated using standardized quantitative assays, mechanistic studies, resistance-development experiments, and safety testing. Strong activity in a diffusion assay or a single MIC experiment alone is insufficient to establish practical antimicrobial potential.

### 5.3. Antihypertensive Activity

Antihypertensive peptides are among the most extensively investigated plant-derived bioactive peptides. Their most widely studied mechanism is the inhibition of angiotensin-converting enzyme (ACE), a zinc-dependent peptidase and key component of the renin–angiotensin–aldosterone system (RAAS) [1,14,19,138,139,140]. ACE catalyzes the conversion of angiotensin I into angiotensin II, a potent vasoconstrictor, and contributes to the degradation of bradykinin, a vasodilatory peptide. Consequently, ACE inhibition may reduce angiotensin II formation, limit vasoconstriction, preserve bradykinin activity, and support blood-pressure regulation [138,139,140].

The potency of ACE-inhibitory peptides is influenced by peptide length, amino acid sequence, terminal residues, hydrophobicity, charge distribution, conformational flexibility, and interactions with the catalytic and substrate-binding regions of ACE. Hydrophobic and aromatic residues, particularly when positioned near the C-terminal region, are frequently associated with strong ACE-inhibitory activity. Proline, phenylalanine, tyrosine, tryptophan, leucine, isoleucine, and valine may facilitate interactions with hydrophobic subsites of the ACE-binding pocket [18,26,138,139].

Positively charged residues, particularly lysine and arginine, may also contribute to peptide–ACE recognition through electrostatic and hydrogen-bonding interactions. Nevertheless, the presence of a particular amino acid or residue class alone does not guarantee strong inhibition, because activity depends on sequence order, residue accessibility, peptide conformation, terminal positioning, and the overall geometry of the peptide–enzyme complex [138,139].

Short peptides, including dipeptides and tripeptides, are frequently associated with relatively strong ACE-inhibitory activity because their small size may facilitate access to the enzyme-binding channel. Low-molecular-weight peptides may also have greater potential for gastrointestinal stability and intestinal absorption than larger peptides [20,22,61,63,138,140]. However, molecular size alone is not a reliable predictor of activity or bioavailability. Some longer peptides may adopt favorable binding conformations, whereas highly active short peptides may be degraded, poorly absorbed, or rapidly metabolized under physiological conditions.

ACE inhibition is commonly evaluated using in vitro enzyme assays and expressed as percentage inhibition or IC_50_ values. Comparisons among studies require careful consideration of ACE source, substrate, reaction conditions, peptide purity, concentration units, calculation method, and positive control [138,139]. Hydrolysate-level activity should also be distinguished from the activity of individual purified peptides because the observed inhibition may result from additive, synergistic, or antagonistic effects among several components.

Among sequence-confirmed ACE-inhibitory peptides, the soybean-derived tetrapeptide NDRP (Asn-Asp-Arg-Pro) provides a well-characterized example. Following identification and chemical synthesis, NDRP produced 87.76 ± 0.61% ACE inhibition and an IC_50_ of 148.28 ± 9.83 μg/mL, behaved as a non-competitive inhibitor, remained stable during simulated gastrointestinal digestion, and was subsequently confirmed in the original hydrolysate by targeted MRM analysis [127] (Table 4).

Although ACE inhibition remains the most common screening endpoint, the antihypertensive effects of food- and plant-derived peptides may involve additional mechanisms. These include renin inhibition, modulation of endothelial nitric oxide synthase and nitric oxide production, effects on calcium-dependent vascular responses, reduction in vascular oxidative stress, and attenuation of endothelial inflammation [138,139,140]. Evidence for these complementary pathways remains peptide- and model-dependent and should therefore be demonstrated experimentally rather than inferred from ACE inhibition alone.

In vitro ACE inhibition should not be considered sufficient evidence of physiological antihypertensive efficacy. Before biological or health-related claims are made, promising peptides should be evaluated for stability during gastrointestinal digestion, resistance to intestinal and plasma peptidases, epithelial transport, bioavailability, and activity in endothelial or vascular-cell models [61,62,63,122,138,139,140]. Studies in spontaneously hypertensive or other appropriate animal models can provide evidence of blood-pressure reduction, dose–response relationships, duration of action, and potential mechanisms.

Ultimately, well-designed human clinical trials are required to establish efficacy, effective dose, safety, and interindividual variability. Therefore, evidence should be interpreted hierarchically, progressing from computational prediction and in vitro ACE inhibition to purified-peptide validation, digestion and transport studies, cellular testing, animal experiments, and controlled clinical evaluation [138,139,140].

### 5.4. Antidiabetic Activity

Plant-derived peptides may contribute to glycemic regulation through multiple and potentially complementary mechanisms, including modulation of carbohydrate digestion, incretin metabolism, insulin signaling, glucose transport, oxidative stress, inflammation, and, in some cases, gut-microbiota-related pathways. Nevertheless, most reported plant-derived antidiabetic peptides have been characterized primarily through in vitro enzyme-inhibition assays, and considerably fewer have been validated in cellular, animal, or human studies [37,38,116,141,142].

One major mechanism is the inhibition of α-amylase and α-glucosidase, which participate sequentially in the digestion of starch and the release of absorbable glucose in the gastrointestinal tract [38,118,141,142]. α-Amylase hydrolyzes internal α-1,4-glycosidic bonds in starch, whereas α-glucosidase catalyzes the final release of glucose from oligosaccharides and disaccharides at the intestinal brush border. By slowing these enzymatic processes, inhibitory peptides may attenuate the rate of carbohydrate digestion and reduce postprandial glucose excursions.

However, excessive α-amylase inhibition may be undesirable because incomplete starch digestion can increase the delivery of fermentable carbohydrates to the colon and contribute to gastrointestinal discomfort. Therefore, a moderate inhibitory effect on α-amylase combined with effective α-glucosidase inhibition may be more relevant for functional-food applications than indiscriminate inhibition of both enzymes [116,141]. Comparisons among studies should also account for enzyme source, substrate, assay conditions, peptide purity, concentration units, positive controls, and the method used to calculate IC_50_ values.

Another important target is dipeptidyl peptidase-IV (DPP-IV), a serine protease involved in the rapid degradation of incretin hormones, particularly glucagon-like peptide-1 and glucose-dependent insulinotropic polypeptide [116,141,142]. Inhibition of DPP-IV can prolong incretin activity, thereby supporting glucose-dependent insulin secretion, limiting inappropriate glucagon release, and contributing to postprandial glucose regulation.

The DPP-IV-inhibitory potency of peptides depends on peptide length, sequence, terminal residues, hydrophobicity, charge distribution, and compatibility with the enzyme-binding pocket [116,141,142]. Proline or alanine near the N-terminal region is frequently associated with DPP-IV recognition because the enzyme preferentially cleaves substrates containing these residues at the penultimate N-terminal position. However, potent DPP-IV-inhibitory peptides do not necessarily reproduce the exact sequence pattern of natural substrates, and inhibitory behavior cannot be predicted from the presence of proline or alanine alone.

Hydrophobic and aromatic residues may enhance interactions with nonpolar regions of the DPP-IV catalytic pocket, whereas charged or hydrogen-bond-forming residues may stabilize peptide–enzyme interactions [116,141,142]. Nevertheless, sequence order, terminal positioning, conformational flexibility, peptide size, and the distinction between true substrates and competitive or noncompetitive inhibitors are also important. Therefore, structure–activity relationships should be confirmed using purified or synthesized peptides, enzyme kinetics, and, where possible, molecular-interaction studies.

Fermented soybean (Douchi) provides an example in which sequence-specific activity has been followed beyond an enzyme-based assay. The peptides VY (Val-Tyr) and SFLLR (Ser-Phe-Leu-Leu-Arg) showed gastrointestinal stability or generated active digestion products and were transported across Caco-2 monolayers [20]. In high-fat-diet-induced insulin-resistant mice, administration at 10 or 50 mg/kg for 8 weeks improved glucose-regulatory outcomes, with particularly notable effects reported for SFLLR [20,128] (Table 4). These findings illustrate the value of linking peptide identification with digestion, intestinal transport, and in vivo validation.

Beyond digestive-enzyme and DPP-IV inhibition, selected plant-derived peptides or hydrolysates may influence insulin sensitivity, glucose uptake, pancreatic β-cell protection, oxidative stress, inflammatory signaling, and pathways involved in glucose and lipid metabolism [141,142,143]. Proposed cellular mechanisms include modulation of insulin-receptor signaling, phosphoinositide 3-kinase/protein kinase B pathways, glucose-transporter translocation, antioxidant defenses, and inflammatory mediators. However, evidence for these mechanisms is highly peptide-, dose-, and model-dependent and should not be inferred solely from enzyme-inhibition results.

In vitro enzyme inhibition alone is insufficient to demonstrate antidiabetic efficacy. Promising peptides should be evaluated for stability during gastrointestinal digestion, susceptibility to intestinal and plasma peptidases, epithelial transport, bioavailability, cytotoxicity, and activity in appropriate intestinal, hepatic, muscle, adipocyte, or pancreatic β-cell models [116,118,141,142]. Animal studies can further assess oral glucose tolerance, fasting glucose, insulin sensitivity, inflammatory status, oxidative stress, and dose–response relationships.

Ultimately, controlled human studies are required to establish whether plant-derived peptides can produce clinically meaningful improvements in postprandial glycemia, insulin sensitivity, or long-term glycemic control. Accordingly, evidence should be interpreted hierarchically, progressing from enzyme inhibition and computational prediction to purified-peptide validation, digestion and transport studies, cellular assays, animal experiments, and clinical evaluation.

### 5.5. Anti-Inflammatory and Immunomodulatory Activity

Chronic, unresolved inflammation contributes to the development and progression of metabolic, cardiovascular, neurodegenerative, autoimmune, and cancer-related disorders. Plant-derived peptides may modulate inflammatory responses by influencing cytokine production, macrophage activation, nitric oxide synthesis, oxidative stress, epithelial-barrier function, and intracellular signaling pathways [1,2,3,143,144,145].

Anti-inflammatory peptides may suppress the production or expression of pro-inflammatory mediators, including tumor necrosis factor-α (TNF-α), interleukin-1β (IL-1β), interleukin-6 (IL-6), inducible nitric oxide synthase (iNOS), cyclooxygenase-2 (COX-2), nitric oxide, and prostaglandin E_2_ (PGE_2_) [143,145]. However, changes in these mediators should be interpreted together with cell viability because reduced cytokine or nitric oxide production may result from cytotoxicity rather than from a specific anti-inflammatory mechanism.

At the molecular level, the reported effects of plant-derived anti-inflammatory peptides are frequently associated with modulation of the nuclear factor-κB (NF-κB) and mitogen-activated protein kinase (MAPK) pathways [143,144]. NF-κB regulates the transcription of numerous inflammatory genes and can be activated by microbial components, cytokines, reactive oxygen species, and cellular stress signals. In its inactive state, NF-κB is retained in the cytoplasm through interaction with inhibitory IκB proteins.

Selected peptides have been reported to reduce IκB phosphorylation or degradation and thereby limit the nuclear translocation of NF-κB subunits, including p65. This may subsequently decrease the expression of inflammatory cytokines and enzymes such as iNOS and COX-2 [143,144]. Nevertheless, NF-κB pathway inhibition should be demonstrated using appropriate measurements, such as IκB phosphorylation or degradation, p65 phosphorylation, nuclear translocation, DNA-binding activity, or target-gene expression, rather than inferred solely from reduced cytokine concentrations.

Plant-derived peptides may also modulate upstream or parallel MAPK signaling pathways, including extracellular signal-regulated kinase, c-Jun N-terminal kinase, and p38 MAPK. These kinases contribute to the transcriptional regulation of inflammatory mediators and cellular stress responses. Peptide-mediated reductions in ERK, JNK, or p38 phosphorylation have been reported in several experimental systems, although the affected pathway and magnitude of the response depend on peptide sequence, concentration, cell type, inflammatory stimulus, and exposure time [144,145].

The anti-inflammatory effects of some peptides may additionally be linked to attenuation of oxidative stress. Reduced intracellular ROS production or activation of endogenous antioxidant defenses may limit redox-sensitive NF-κB and MAPK signaling and thereby decrease inflammatory mediator production [129,130,143,144]. However, antioxidant and anti-inflammatory activities should be evaluated independently because chemical radical-scavenging activity alone does not establish modulation of intracellular inflammatory pathways.

Immunomodulatory activity is broader than anti-inflammatory activity and may involve either suppression of excessive immune activation or enhancement of protective immune responses. Depending on their sequence, concentration, target cells, and biological context, peptides may influence phagocytosis, immune-cell proliferation, cytokine balance, macrophage activation and polarization, natural-killer-cell activity, epithelial-barrier integrity, and interactions between immune cells, intestinal epithelial cells, and the gut microbiota [144,145].

Macrophage polarization is frequently evaluated using changes in pro-inflammatory and resolution-associated markers. Nevertheless, the simplified M1/M2 classification does not fully represent the diversity and plasticity of macrophage phenotypes in tissues. Therefore, conclusions regarding macrophage polarization should ideally be based on multiple phenotypic, transcriptional, and functional markers rather than on a single cytokine or surface marker.

Because immune responses are highly context-dependent, evaluation of anti-inflammatory and immunomodulatory peptides requires carefully selected experimental models. Common approaches include lipopolysaccharide-stimulated macrophages, intestinal epithelial and immune-cell cocultures, cytokine-induced inflammatory models, and disease-relevant animal systems [143,144]. The selected stimulus, cell origin, peptide concentration, treatment sequence, positive control, and exposure duration should be clearly reported.

Cytotoxicity, endotoxin contamination, peptide purity, dose–response behavior, and stability in cell-culture media should also be assessed.

A relevant example is the *Vicia faba*-derived peptide TIKIPAGT, which was initially prioritized using machine-learning analysis and subsequently validated experimentally. The peptide reduced TNF-α secretion in vitro, survived upper gastrointestinal digestion, crossed an intestinal barrier model, and demonstrated notable stability in human plasma [21]. This progression from computational prediction to cellular activity and stability assessment provides a stronger level of evidence than prediction or hydrolysate-level activity alone (Table 4). Endotoxin contamination is particularly important in immunological assays because even low levels of bacterial lipopolysaccharide may independently alter cytokine production and macrophage activation.

Ultimately, reductions in inflammatory markers in a simplified cell model should not be interpreted as evidence of therapeutic efficacy. Promising peptides require confirmation of gastrointestinal stability, intestinal transport, bioavailability, safety, and efficacy in relevant in vivo systems. Human studies are necessary before clinically meaningful anti-inflammatory or immunomodulatory effects can be established.

### 5.6. Other Regulatory Activities

In addition to antioxidant, antimicrobial, antihypertensive, antidiabetic, and anti-inflammatory effects, plant-derived peptides may exhibit several other regulatory activities, including hypocholesterolemic, anti-obesity, opioid-like, mineral-binding, and anticancer-related effects [1,2,3,14,17,32,33,34,41,51,52,53,54,55,56,57,58,59,146,147,148,149]. However, the strength of evidence differs substantially among these activities, and many proposed effects remain based primarily on enzyme assays, cell-free systems, or experimental models.

Hypocholesterolemic peptides may influence cholesterol homeostasis through several gastrointestinal and hepatic mechanisms. In the intestinal lumen, selected peptides or peptide-rich hydrolysates may bind bile acids, interfere with cholesterol incorporation into mixed micelles, inhibit cholesterol esterase, or increase fecal bile-acid excretion [14,33,147]. Increased loss of bile acids may promote hepatic conversion of cholesterol into new bile acids, thereby contributing to a reduction in circulating cholesterol. Some peptides may also influence hepatic cholesterol metabolism by modulating enzymes, transporters, or transcriptional pathways involved in cholesterol synthesis and clearance [14,146].

Nevertheless, bile-acid binding or inhibition of cholesterol micellization in vitro does not necessarily demonstrate a cholesterol-lowering effect in vivo. Physiological relevance depends on peptide stability during digestion, persistence within the intestinal lumen, effective concentration, interactions with dietary lipids and bile salts, and confirmation in animal or human studies.

Anti-obesity peptides may act by inhibiting pancreatic lipase, decreasing the digestion and intestinal absorption of dietary triacylglycerols, modulating adipocyte differentiation, influencing adipokine secretion, or regulating pathways involved in lipid accumulation and energy metabolism [14,41,146]. Some peptides or hydrolysates have also been associated with changes in fatty-acid oxidation, thermogenesis, satiety-related signaling, or gut-microbiota composition. However, these effects are peptide-, dose-, and model-dependent and should not be inferred solely from pancreatic-lipase inhibition.

Opioid-like peptides, also referred to as food-derived exorphins, may be released from dietary proteins during gastrointestinal digestion or fermentation and interact with opioid receptors [147]. Certain cereal-derived peptides, particularly gluten-derived exorphins, have been reported to influence gastrointestinal motility, secretion, pain-related signaling, and neuroendocrine responses. However, their physiological importance depends on resistance to gastrointestinal degradation, receptor selectivity, local concentration, epithelial transport, and access to peripheral or central target tissues [147]. Therefore, receptor-binding or opioid activity observed in vitro should not be interpreted as evidence of systemic neurological effects without appropriate pharmacokinetic and in vivo confirmation.

Metal-chelating peptides can coordinate mineral ions through carboxyl, amino, imidazole, sulfhydryl, phosphate, or backbone carbonyl groups. Aspartic acid, glutamic acid, histidine, cysteine, serine, and phosphorylated residues may contribute to mineral coordination, depending on their sequence position and spatial arrangement [3,26,148]. Peptide complexes with iron, calcium, zinc, copper, or magnesium may improve mineral solubility, protect minerals against precipitation or antagonistic dietary components, and potentially enhance intestinal availability [148].

Metal chelation may also reduce transition-metal-catalyzed radical formation, particularly when peptides bind redox-active iron or copper. However, strong chelation does not automatically improve mineral bioavailability because excessive binding may limit mineral release and epithelial uptake. Consequently, peptide–mineral complexes should be assessed for binding stability, mineral release during simulated digestion, intestinal transport, and bioavailability rather than by chelation capacity alone [148].

Some plant-derived peptides have demonstrated anticancer-related effects in vitro, including inhibition of cell proliferation, induction of apoptosis, disruption of cancer-cell membranes, modulation of oxidative stress, cell-cycle arrest, and interference with migration, invasion, or angiogenesis-related pathways [2,3,149]. Proposed mechanisms include mitochondrial dysfunction, caspase activation, regulation of pro- and anti-apoptotic proteins, modulation of tumor-associated signaling pathways, and selective interactions with the comparatively anionic membranes of some cancer cells [149].

Lunasin, a 43-amino-acid soybean peptide associated with anti-inflammatory and anticancer-related activities, also provides one of the relatively uncommon examples for which human absorption has been investigated. In five healthy men consuming 50 g of soy protein daily for 5 days, plasma lunasin concentrations averaged 66.0 ± 25.4 ng/mL at 30 min and 71.0 ± 32.8 ng/mL at 1 h, with an estimated mean absorption of approximately 4.5%; lunasin-related sequences were detected by MALDI-TOF and LC–MS/MS [22]. Importantly, these findings demonstrate human absorption/bioavailability rather than clinical efficacy.

Nevertheless, anticancer-related claims require particularly careful interpretation. Reduced cancer-cell viability may result from nonspecific cytotoxicity, assay interference, peptide aggregation, or unusually high experimental concentrations. Promising peptides should therefore be evaluated in parallel against non-transformed cells, and their selectivity index, mechanism of cell death, metabolic and serum stability, effective concentration, and delivery to tumor tissue should be determined [149].

Confirmation in relevant three-dimensional cell models, organoids, animal tumor models, pharmacokinetic studies, and ultimately clinical trials is required before plant-derived peptides can be considered anticancer candidates. Accordingly, activity observed only in a cancer-cell proliferation assay should be described as in vitro anticancer-related or cytotoxic activity rather than as established anticancer efficacy.

### 5.7. Structure–Activity Relationships of Bioactive Peptides

Understanding structure–activity relationships is essential for predicting, prioritizing, and optimizing the biological functions of plant-derived peptides. Several recurring structural trends have been identified; however, no single physicochemical feature or amino acid motif can explain all peptide activities across different targets, experimental systems, and biological environments.

Accordingly, physicochemical descriptors such as peptide length, molecular weight, net charge, hydrophobicity, amphipathicity, or the presence of particular amino acid residues should be regarded as contributory features rather than independent predictors of biological activity. Their effects are strongly context-dependent and are influenced by sequence order, residue position, terminal composition, three-dimensional conformation, target accessibility, proteolytic stability, concentration, and the experimental environment. Therefore, similar values for molecular weight, charge, or hydrophobicity may be associated with markedly different biological activities, whereas peptides with distinct physicochemical profiles may interact with the same biological target through different structural mechanisms [1,2,3,18,23,26,146,150,151].

Peptide molecular size can influence target accessibility, gastrointestinal stability, intestinal transport, and mechanism of action. Low-molecular-weight peptides, particularly dipeptides and tripeptides, may access relatively narrow enzyme-binding pockets and may have greater potential for transport through peptide transporters or paracellular pathways [20,21,22,51,52,53,54,118,146]. Nevertheless, low molecular weight alone does not guarantee high bioactivity or bioavailability. Some larger peptides require sufficient length to adopt amphipathic secondary structures, interact with membranes, engage multiple binding subsites, or display immunomodulatory activity.

Hydrophobicity can enhance interactions with lipid bilayers and nonpolar regions of enzymes, receptors, or other molecular targets. Moderate hydrophobicity is therefore frequently associated with antimicrobial, antioxidant, ACE-inhibitory, and other biological activities [1,3,26,28,146]. However, excessive hydrophobicity may reduce aqueous solubility, promote peptide aggregation, increase bitterness, enhance nonspecific membrane interactions, and increase hemolytic or cytotoxic effects. The influence of hydrophobicity must consequently be considered together with peptide charge, amphipathicity, sequence order, and conformational state.

Positive net charge is particularly important for many membrane-active antimicrobial peptides because it facilitates electrostatic attraction to negatively charged microbial cell-envelope components. However, antimicrobial activity and selectivity depend not only on the total number of cationic residues but also on their spatial distribution relative to hydrophobic residues, the resulting amphipathic structure, peptide flexibility, membrane composition, and ionic conditions [28,132,133,134]. Excessive positive charge may also increase nonspecific binding to host cells or biological macromolecules.

The biological contribution of individual amino acids is strongly dependent on their position within the peptide sequence. For ACE inhibition, hydrophobic, aromatic, or cyclic residues located near the C-terminal region are frequently associated with favorable binding to hydrophobic subsites of the ACE active pocket [138,139,150]. Proline and aromatic residues at or near the C-terminus are common among potent short ACE-inhibitory peptides, but they should be regarded as recurrent structural tendencies rather than universal requirements. Peptide length, N-terminal composition, charge, conformational flexibility, and the overall geometry of the peptide–ACE complex also influence inhibitory potency. Published quantitative SAR studies confirm that residue position and side-chain physicochemical characteristics are critical for short ACE-inhibitory peptides. For DPP-IV inhibition, peptides containing proline or alanine at the second position from the N-terminus are frequently associated with DPP-IV recognition because the enzyme naturally cleaves substrates with Pro or Ala in this position [116,141,142,151]. Nevertheless, DPP-IV inhibitors may function as substrates, competitive inhibitors, or inhibitors with alternative binding modes, and potent inhibitory activity cannot be predicted solely from an N-terminal Pro/Ala motif. Hydrophobic, aromatic, charged, and hydrogen-bond-forming residues elsewhere in the sequence may also influence binding and inhibition.

For antimicrobial activity, the amphipathic spatial distribution of cationic and hydrophobic residues is generally more informative than bulk amino acid composition. Peptides with similar charge and hydrophobicity may display different antimicrobial and hemolytic activities because of differences in secondary structure, residue distribution, aggregation behavior, membrane affinity, and conformational adaptability [28,135,137]. Therefore, descriptors such as hydrophobic moment, charge density, amphipathicity, helicity, and membrane selectivity should be evaluated together.

For antioxidant activity, aromatic residues such as tyrosine and tryptophan may participate in electron or hydrogen-atom donation and stabilization of radical intermediates. Methionine and cysteine may contribute through oxidation–reduction reactions, whereas histidine may support proton transfer or metal coordination. Proline and hydrophobic residues may facilitate interactions with lipid phases and thereby enhance protection against lipid oxidation [26,27,115,130,146]. However, the antioxidant contribution of these residues depends on their sequence position, solvent accessibility, neighboring amino acids, terminal location, peptide conformation, and the specific assay system. Reordering the same amino acid composition can substantially alter antioxidant activity.

Peptide function therefore cannot be predicted reliably from amino acid composition alone. Sequence order, residue position, terminal composition, three-dimensional conformation, charge distribution, amphipathicity, molecular flexibility, aggregation tendency, protease susceptibility, and stability in biological media must also be considered [1,3,146,150,151]. Post-translational or chemical modifications may further alter peptide charge, hydrophobicity, conformation, enzymatic stability, and target affinity.

The apparent activity of a peptide may also differ markedly among purified enzyme systems, chemical assays, complex hydrolysates, cellular environments, and in vivo models. In crude hydrolysates, measured activity may reflect additive, synergistic, or antagonistic interactions among multiple peptides and residual non-peptide components. In cellular and physiological environments, peptide degradation, binding to proteins, matrix interactions, transport limitations, metabolism, and target accessibility may substantially alter activity.

Accordingly, structure–activity relationships derived from peptide databases, molecular docking, quantitative SAR models, or machine-learning algorithms should be treated as tools for hypothesis generation and candidate prioritization rather than as definitive proof of biological function. Reliable SAR analysis requires experimental comparison of purified or chemically synthesized peptide analogues, systematic residue substitution, truncation or sequence-scrambling studies, quantitative activity assays, biophysical interaction measurements, and validation in relevant cellular or in vivo systems [10,119,120,121,122,146,150,151].

### 5.8. Computational Prediction and In Silico Screening

Computational approaches have become indispensable tools for accelerating the discovery and rational design of plant-derived bioactive peptides. Compared with conventional bioactivity-guided purification, in silico methods substantially reduce the time, cost, and experimental workload required to identify promising peptide candidates by prioritizing protein sources, hydrolysis strategies, peptide sequences, and biological targets before laboratory validation. Rather than replacing experimental research, computational methods serve as hypothesis-generating and prioritization tools that complement biochemical, analytical, and biological validation [10,119,120,121,122,152].

Sequence-based prediction algorithms analyze peptide sequences using physicochemical and structural descriptors, including molecular weight, amino acid composition, sequence motifs, hydrophobicity, net charge, theoretical isoelectric point, amphipathicity, hydrophobic moment, aliphatic index, instability index, and grand average of hydropathicity (GRAVY). These descriptors are subsequently incorporated into statistical and machine-learning models to predict the likelihood that a peptide possesses a particular biological activity [10,121,122,152,153,154].

Modern computational platforms employ machine-learning algorithms such as support vector machines, random forests, artificial neural networks, gradient boosting, and, increasingly, deep-learning architectures. Depending on the training dataset, these models have been developed to predict antimicrobial, antioxidant, antihypertensive, antidiabetic, anti-inflammatory, anticancer, toxic, allergenic, hemolytic, and cell-penetrating properties directly from peptide sequences [121,122,153,155].

General bioactivity-ranking algorithms estimate the probability that a peptide exhibits biological activity without assigning a specific mechanism, whereas specialized prediction platforms are trained to recognize defined peptide classes, such as antimicrobial peptides, toxic peptides, or allergenic proteins. Consequently, predictions generated by different computational tools should not be considered interchangeable because they rely on different datasets, feature-selection strategies, machine-learning architectures, and prediction endpoints [121,153,154,155,156]. Representative computational databases and tools used for the identification, prediction, and molecular evaluation of plant-derived bioactive peptides are summarized in Table 5.

In practical terms, tool selection should be matched to the specific research question. BIOPEP-UWM is useful for identifying previously reported bioactive sequences and simulating enzyme-specific release from parent proteins, whereas PeptideRanker can be used for preliminary ranking of general bioactivity. For antimicrobial candidates, specialized resources such as CAMPR4 and DBAASP are more appropriate than general bioactivity predictors. Safety-related screening can be performed using tools such as ToxinPred 3.0, AllerTOP v2.0, and HemoPI for predicted toxicity, allergenicity, and hemolytic potential, respectively, while ExPASy ProtParam provides basic sequence-derived physicochemical descriptors. When a defined molecular target is available, AutoDock Vina can be used to generate candidate binding poses, followed by GROMACS molecular-dynamics simulations to examine the stability and dynamics of selected complexes. Importantly, these tools should be used sequentially and complementarily rather than as interchangeable predictors, and no computational output should be considered evidence of biological efficacy without experimental confirmation [10,119,152,153,154,155,156,157,158,159,160,161,162].

### 5.9. Molecular Docking and Molecular Dynamics Simulations

**Figure 2:**
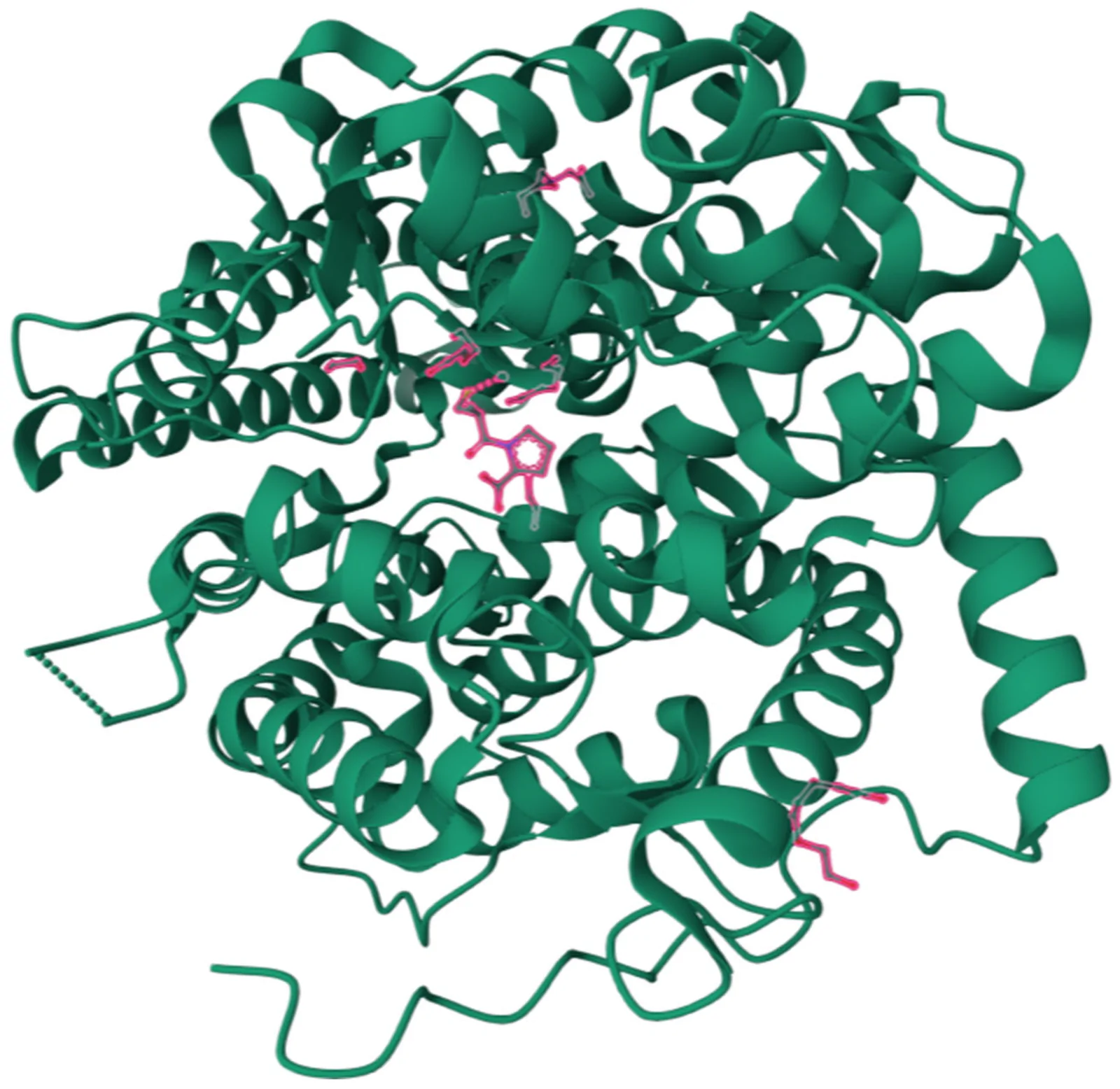
Structural representation of the ACE active site based on an ACE–captopril crystal structure. Captopril (pink) is shown as a reference inhibitor to illustrate the catalytic binding region relevant to ACE-inhibitory plant-derived peptides. The structure is freely available from the RCSB Protein Data Bank (https://www.rcsb.org/3d-view/9SS9/1; accessed on 19 August 2026) and corresponds to PDB ID 9SS9 [163]. The figure is intended to provide structural context for the ACE catalytic site and its relevance to the inhibitory activity of plant-derived peptides, rather than to depict a specific plant peptide–ACE complex.

Molecular docking is widely used to predict possible binding conformations and interaction patterns between plant-derived peptides and biological targets, including angiotensin-converting enzyme (ACE, Figure 2), renin, DPP-IV, α-glucosidase, α-amylase, cyclooxygenase-2, lipoxygenase, acetylcholinesterase, tyrosinase, and microbial enzymes [10,119,160,162].

Docking algorithms generate candidate peptide–target poses and rank them using approximate scoring functions based on steric complementarity, hydrogen bonding, hydrophobic contacts, electrostatic interactions, salt bridges, aromatic interactions, and, where relevant, metal coordination.

Docking may provide mechanistic hypotheses regarding the residues and subsites involved in peptide recognition. For ACE-inhibitory peptides, it can identify interactions with the S1, S1′, and S2 subsites, catalytic residues, and the zinc-containing region of the active site [138,139,160,162]. For DPP-IV inhibitors, docking may indicate whether a peptide occupies the catalytic cavity or interacts with residues involved in substrate recognition, whereas docking to α-glucosidase or α-amylase may suggest whether peptide binding interferes with substrate access or catalytic-site organization [116,141,142,162].

Docking results should nevertheless be interpreted cautiously. Peptides are generally larger and more conformationally flexible than conventional small-molecule ligands, and their binding may involve extensive rearrangement of both the peptide and receptor. Many docking protocols incompletely sample peptide conformations, receptor flexibility, solvent effects, protonation states, and induced-fit behavior [160,162]. Consequently, a favorable docking score or visually plausible binding pose should not be regarded as direct evidence of binding affinity, enzyme inhibition, or biological activity.

The identification of hydrogen bonds or other contacts in a docking pose is also insufficient to establish that these interactions remain stable under dynamic and solvated conditions. Docking scores produced by different software packages or scoring functions are not necessarily comparable, and small numerical differences between peptides may not be biologically meaningful [10,160,161,162]. Where possible, docking protocols should be validated by redocking a known ligand or peptide, reproducing an experimentally determined binding pose, and comparing computational rankings with experimentally measured activity.

Molecular dynamics simulations complement static docking studies by evaluating the time-dependent stability, conformational flexibility, and interaction behavior of peptide–target complexes in an explicitly or implicitly solvated and ionized environment [161,162]. Common trajectory analyses include root-mean-square deviation, root-mean-square fluctuation, radius of gyration, solvent-accessible surface area, hydrogen-bond occupancy, inter-residue distances, secondary-structure changes, and interaction-energy fluctuations.

Molecular dynamics may reveal whether a docked peptide remains within the proposed binding region, whether key contacts persist, and whether binding induces conformational changes in the peptide or target protein. However, apparent stability during a simulation does not independently prove biological binding because results depend on the initial pose, force field, protonation states, solvent and ion composition, boundary conditions, equilibration procedure, trajectory length, and adequacy of conformational sampling [161,162].

End-point binding free-energy methods, particularly MM-PBSA and MM-GBSA, are frequently applied to molecular-dynamics trajectories to estimate relative peptide–target interaction energies and identify favorable energetic contributions [164]. These approaches combine molecular-mechanics energies with continuum-solvation and solvent-accessible-surface-area terms and may be useful for comparing structurally related candidates under a consistent computational protocol.

Nevertheless, MM-PBSA and MM-GBSA values are approximations rather than experimentally equivalent binding free energies. Their accuracy can be affected by trajectory sampling, dielectric constants, treatment of entropy, conformational reorganization, solvation models, and the highly charged nature of many peptides [164]. Therefore, these methods are generally more appropriate for supportive interpretation or relative ranking within a closely related peptide series than for reporting absolute binding affinity.

For antimicrobial peptides acting primarily through membrane disruption, docking to an isolated intracellular protein may not reproduce the dominant mechanism of action. Lipid-bilayer molecular dynamics simulations are more suitable for investigating peptide adsorption, orientation, folding, insertion, aggregation, membrane thinning, lipid disorder, pore formation, and translocation [165]. The selected membrane model should reflect the target organism because bacterial and mammalian membranes differ in lipid composition, surface charge, cholesterol content, and biophysical properties.

Both all-atom and coarse-grained molecular dynamics can be applied to peptide–membrane systems. All-atom simulations provide detailed information on residue–lipid and water-mediated interactions, whereas coarse-grained simulations allow larger systems and longer effective time scales to be explored [161,165]. However, spontaneous peptide insertion or pore formation may occur on time scales that remain difficult to sample, and observed behavior can depend strongly on peptide concentration, initial orientation, membrane composition, force field, and simulation duration.

Overall, docking and molecular dynamics should be regarded as complementary hypothesis-generating tools. Docking proposes possible binding arrangements, whereas molecular dynamics evaluates their behavior under simulated dynamic conditions. Reliable conclusions require integration with purified or synthesized peptides, enzyme kinetics, biophysical binding measurements, mutational or residue-substitution studies, membrane assays where appropriate, and cellular or in vivo validation [10,119,162,166].

### 5.10. Machine Learning and Artificial Intelligence in Peptide Discovery

Machine learning and artificial intelligence have emerged as powerful approaches for peptide classification, quantitative structure–activity relationship modeling, activity prediction, candidate prioritization, and de novo peptide design. These methods can identify nonlinear relationships between peptide sequence, physicochemical properties, structural descriptors, and experimentally measured biological activity that may not be apparent through conventional statistical analysis [121,122,167,168].

Commonly applied algorithms include support vector machines, random forests, gradient-boosting methods, artificial neural networks, convolutional neural networks, recurrent neural networks, graph neural networks, and transformer-based architectures. More recent models use protein-language-model embeddings to capture contextual relationships among amino acid residues without relying exclusively on manually selected descriptors [121,122,167,168,169].

Depending on the available training data, machine-learning models may perform binary or multiclass peptide classification, predict quantitative potency, identify multifunctional sequences, or generate new peptide candidates with predefined properties. Multi-label models are particularly relevant because an individual peptide may exhibit more than one biological function, including antioxidant, antimicrobial, antihypertensive, antidiabetic, or anti-inflammatory activity [154,168,169].

In plant peptide research, virtual peptide libraries can be generated from annotated plant proteomes, storage proteins, and proteins recovered from agro-industrial by-products. In silico digestion and sequence-fragmentation strategies can produce large candidate sets that are subsequently ranked according to predicted activity, toxicity, allergenicity, hemolytic potential, solubility, stability, and physicochemical suitability [10,152,156,157,158].

Machine-learning models may also integrate heterogeneous experimental information derived from peptidomics, LC–MS/MS identification, bioactivity assays, molecular docking, and molecular dynamics simulations. Such multimodal approaches may improve candidate prioritization by combining sequence-based predictions with structural, interaction, and experimentally measured features [10,119,167,168,169].

Artificial intelligence may further support de novo peptide generation. Generative and transformer-based models can propose novel sequences optimized for several simultaneous objectives, such as high predicted bioactivity, reduced toxicity, limited hemolysis, improved solubility, and enhanced proteolytic stability [167,168,169]. However, generated sequences may fall outside the chemical and biological space represented in the training data, making experimental confirmation especially important.

Model reliability remains constrained by limited dataset size, class imbalance, sequence redundancy, inconsistent assay protocols, missing negative data, and variability in activity thresholds. Models trained on highly similar sequences may show excellent internal performance while performing poorly on independent or structurally distinct peptide datasets [10,121,167,168,169].

Plant-derived peptides remain underrepresented in many training datasets compared with dairy, animal, microbial, and synthetic peptides. Consequently, predictions obtained for plant storage-protein fragments may fall outside the applicability domain of models constructed primarily from other peptide classes [1,10,119,167,168,169].

Independent external validation, sequence-similarity-aware dataset splitting, transparent reporting of model architecture and hyperparameters, and use of appropriate evaluation metrics are therefore essential. Accuracy alone may be misleading for strongly imbalanced datasets; sensitivity, specificity, precision, F1 score, Matthews correlation coefficient, receiver-operating-characteristic area under the curve, and precision–recall analysis may provide a more complete assessment.

Accordingly, machine-learning and AI predictions should be regarded as hypothesis-generating and candidate-prioritization tools rather than direct evidence of biological function. High-ranking sequences must be confirmed through peptide synthesis or targeted purification, structural characterization, standardized biochemical assays, cytotoxicity and safety testing, and validation in relevant cellular or in vivo systems [10,121,167,168,169].

### 5.11. Integrated In Silico–In Vitro–In Vivo Validation Framework

The most effective strategy for plant-derived peptide discovery relies on iterative integration of computational prediction and experimental validation. In silico digestion, sequence-based prediction, molecular docking, molecular dynamics, and machine-learning models can first be used to screen plant proteomes and prioritize candidates with favorable predicted biological and safety profiles [10,119,152,162,167]. For clarity, the evidence supporting plant-derived peptide bioactivity should be interpreted hierarchically. In silico predictions, including sequence-based screening, molecular docking, molecular dynamics, and AI/ML approaches, represent hypothesis-generating and candidate-prioritization evidence [10,121,122,167,168,169]. Chemical and enzyme-based assays provide initial functional evidence but do not establish activity under biological conditions. Validation using purified or chemically synthesized peptides in quantitative biochemical or biophysical assays provides stronger sequence-specific evidence, while cellular studies further assess activity in a biologically relevant environment [10,119,167,168,169]. Animal experiments can provide evidence of systemic efficacy, dose–response relationships, pharmacokinetic behavior, and safety, whereas well-designed human studies represent the highest level of evidence for physiological or clinical relevance [20,21,22,61,62,63,118]. Accordingly, computational predictions should guide experimental prioritization but should not be interpreted as direct evidence of biological efficacy in the absence of experimental confirmation [10,119,121,122].

Prioritized peptides may then be chemically synthesized, isolated from active fractions, or quantified using targeted mass-spectrometric methods. Their predicted functions should be evaluated using standardized biochemical assays, cell-based models, membrane studies, or molecular-interaction experiments appropriate to the proposed activity.

The resulting experimental data, including IC_50_ values, inhibition constants, minimum inhibitory concentrations, binding affinities, cytotoxicity, hemolysis, solubility, and stability measurements, can subsequently be incorporated into updated computational models. This feedback process may improve model performance within a defined peptide class or botanical source and identify previously unrecognized sequence–activity relationships [121,167,169,170].

Active-learning approaches attempt to select the next peptide candidates that are expected to provide the greatest improvement in predictive accuracy or functional performance. However, active learning does not automatically outperform random experimental selection, particularly when datasets are very small, noisy, heterogeneous, or strongly imbalanced [170]. Its value therefore depends on the quality of uncertainty estimation, the selection strategy, and the experimental endpoint.

An iterative in silico–in vitro workflow can reduce unnecessary synthesis and screening by concentrating experimental resources on candidates with the highest predicted value. This approach is particularly relevant to underutilized crops, regional plant species, and agro-industrial residues, where experimental peptide datasets are often limited and large numbers of potential protein fragments remain unexplored [1,10,167].

The validation loop should not be restricted to positive activity results. Inactive, weakly active, cytotoxic, unstable, or poorly soluble peptides provide valuable negative and quantitative data that can improve model discrimination and reduce selection bias. Standardized reporting and inclusion of experimentally confirmed negative examples are therefore critical for developing reliable predictive models.

Computational screening should be positioned between peptide identification and definitive biological validation. It can guide candidate prioritization, suggest possible targets, and support mechanistic interpretation, but it does not replace direct experimental evidence. The strongest conclusions are obtained when predicted activity is confirmed using purified or synthesized peptides in concentration-dependent assays and supported by orthogonal biochemical or biophysical methods.

Where oral or systemic activity is proposed, the validation sequence should additionally include simulated gastrointestinal digestion, brush-border or plasma stability, intestinal transport models, relevant cellular assays, and, where justified, animal or human studies [20,21,22,61,62,63,64,118]. Thus, the integrated workflow should progress from computational probability to analytical confirmation, biological activity, mechanistic evidence, and physiological validation. Importantly, this framework should be viewed as an iterative cycle rather than a strictly linear sequence. Data obtained from biochemical assays, cellular models, gastrointestinal stability and transport studies, animal experiments, and, when available, human studies should be fed back into computational models and subsequent experimental design. Both positive and negative experimental outcomes can refine sequence–activity relationships, improve model applicability, identify limitations in predicted candidates, and guide the next round of peptide selection. Accordingly, the in silico → in vitro → in vivo cycle provides a continuous framework for moving from prediction to physiological validation and back to improved candidate prioritization [10,121,167,168,169,170]. A practical validation sequence can therefore be defined according to the computational endpoint. Predicted peptide sequences should first be confirmed analytically by LC–MS/MS or obtained as purified or chemically synthesized peptides of defined identity and purity. Predicted enzyme inhibitors should then be evaluated using concentration-dependent inhibition and kinetic assays; predicted antimicrobial peptides should be tested by standardized MIC-based methods together with membrane and cytotoxicity assays; predicted peptide–target interactions should be examined using appropriate biophysical or mutational approaches; and predicted toxicity, hemolysis, or allergenicity should be assessed using dedicated experimental safety assays. Candidates intended for oral application should additionally undergo gastrointestinal-stability and intestinal-transport testing before animal or human evaluation. Thus, each computational prediction should be linked to an experimentally measurable endpoint rather than validated solely by a second computational method.

### 5.12. Challenges, Limitations and Future Perspectives

Despite substantial progress in the biological and computational characterization of plant-derived peptides, several major translational challenges remain. High activity in chemical, enzyme-based, or simplified cellular assays does not necessarily translate into physiological efficacy because peptides may be degraded during gastrointestinal digestion, hydrolyzed by brush-border or plasma peptidases, poorly absorbed, rapidly metabolized, or unable to reach target tissues at biologically effective concentrations [4,20,21,22,61,62,63,64,118,140,171,172].

Bioavailability is influenced by peptide length, sequence, charge, hydrophobicity, aggregation, interactions with food-matrix components, and susceptibility to enzymatic degradation. Strategies such as targeted hydrolysis, structural modification, encapsulation, lipid-based delivery systems, nanoparticles, liposomes, emulsions, and other protective carrier systems may improve peptide stability, intestinal transport, and delivery. However, these approaches require independent evaluation of their safety, efficacy, physicochemical stability, and compatibility with the intended food or nutraceutical matrix [68,69,118,172].

A second challenge is that crude plant protein hydrolysates or peptide fractions may exhibit stronger activity than isolated peptides. Such effects may result from additive or synergistic interactions among multiple peptide species or from the presence of residual non-peptide constituents. Purification may therefore reduce the measured activity even when individual active sequences are successfully identified [104,105,108,171].

This complexity complicates standardization, dose definition, mechanism-of-action studies, and quality control. Studies should clearly distinguish among the activity of a crude hydrolysate, a molecular-weight fraction, a chromatographic fraction, and an individual purified or chemically synthesized peptide. The identity, purity, concentration, and compositional characteristics of the tested material should therefore be reported in sufficient detail [104,105,108,120].

A third limitation is the underrepresentation of plant-derived sequences in peptide databases and machine-learning datasets. Many predictive models are dominated by dairy, animal, microbial, therapeutic, or synthetic peptide sequences, which may limit their ability to generalize to plant storage-protein fragments, unusual plant peptides, and peptides generated through fermentation, germination, or controlled enzymatic hydrolysis [10,119,121,152,153,154,155,156,157,158,167,168,169,170].

Incomplete annotation of non-model plant proteomes further limits database-assisted peptide identification and in silico digestion. High-quality plant-specific databases should therefore include experimentally confirmed peptide sequences, parent proteins, botanical sources, production conditions, quantitative activity data, inactive or weakly active controls, gastrointestinal stability, transport, toxicity, and allergenicity information [10,119,121,152,153,154,155,156,157,158].

A fourth challenge is the lack of methodological harmonization. Differences in enzyme source, substrate composition, peptide purity, concentration units, reaction time, pH, positive and negative controls, assay calculation, cellular models, and reporting formats make direct comparisons among studies difficult [95,98,99,100,115,116,117,120,171].

Future studies should adopt standardized gastrointestinal digestion protocols, validated biochemical and cellular assays, appropriate biological and technical replicates, transparent statistical analyses, and detailed reporting of peptide identity, purity, concentration, and dose–response relationships. The use of reference peptides, standardized positive and negative controls, and harmonized activity-reporting units would further improve interlaboratory reproducibility and comparability [98,99,100,109,110,111,112,115,116,117,118,119,120].

Future research should also prioritize intestinal transport, systemic stability, pharmacokinetics, dose–response relationships, chronic safety, allergenicity, and efficacy in physiologically relevant models. Caco-2 monolayers, advanced intestinal cell systems, organoids, tissue-based models, and appropriately designed animal studies may help bridge the gap between simplified in vitro assays and clinical investigation [61,62,63,64,118,140,171,172].

Greater integration of high-resolution peptidomics, ion-mobility mass spectrometry, molecular docking, molecular dynamics, machine learning, multi-omics, toxicology, allergenicity assessment, formulation science, and translational biology will be essential for identifying plant-derived peptides with genuine physiological relevance [9,10,109,110,111,112,113,119,121,162,166,167,168,169,170,171,172]. Computational and experimental approaches should be developed as components of an iterative validation system rather than as independent stages.

#### 5.12.1. Regulatory Frameworks and Health Claims

Regulatory requirements for plant-derived peptide ingredients depend on the jurisdiction, intended use, manufacturing process, history of consumption, product category, and claims proposed for the final product. In the European Union, an ingredient without a significant history of human consumption within the Union before 15 May 1997 may fall within the scope of Regulation (EU) 2015/2283 on novel foods and may require pre-market authorization [173]. Safety assessment considers the identity and composition of the ingredient, manufacturing process, proposed uses and anticipated exposure, nutritional characteristics, toxicological information, and allergenic potential [173].

Health claims are regulated separately under Regulation (EC) No 1924/2006 [174]. EFSA assessment requires adequate characterization of the food or food constituent, demonstration that the claimed effect is beneficial to human health, and sufficient scientific evidence supporting a cause-and-effect relationship. Well-designed human intervention studies are particularly important when specific physiological health benefits are claimed [175].

In the United States, plant-derived protein hydrolysates or peptide ingredients intended for use in conventional foods must comply with the applicable food-ingredient requirements. An intended use may qualify as generally recognized as safe (GRAS) when safety under the proposed conditions of use is generally recognized among appropriately qualified experts [176]. A manufacturer may submit a GRAS notice to the U.S. Food and Drug Administration. However, an FDA response stating that the agency has “no questions” regarding the notifier’s GRAS conclusion should not be interpreted as approval of therapeutic efficacy or of a health claim. Health claims, qualified health claims, and structure/function claims are subject to separate regulatory requirements and different standards of scientific substantiation [176,177,178]. Consequently, regulatory status in one jurisdiction cannot automatically be transferred to another, and the development strategy should be adapted to the intended market, product category, conditions of use, and proposed claims.

#### 5.12.2. Allergenicity, Toxicological Evaluation and Human Evidence

Safety evaluation should accompany efficacy assessment throughout peptide development. Allergenicity assessment can follow a tiered approach incorporating the botanical source and known allergen history, sequence comparison with characterized allergens, protein and peptide profiling, gastrointestinal and thermal stability, and, where appropriate, IgE-binding, basophil-activation, cellular, or in vivo studies [71,72,73,157,173]. Although enzymatic hydrolysis may reduce allergenicity by disrupting conformational or linear IgE-reactive epitopes, residual or newly exposed reactive fragments may remain. Reduced molecular size should therefore not automatically be interpreted as absence of allergenic risk.

Toxicological evaluation should be adapted to the nature of the ingredient, intended dose, duration of exposure, manufacturing process, and existing history of safe use. Relevant assessments may include cytotoxicity, genotoxicity, repeated-dose toxicity, microbiological safety, contaminants, antinutritional factors, potential adverse interactions, and dose–response relationships [173].

Human intervention studies become particularly important when specific physiological or health claims are proposed. Such studies should use adequately characterized test materials, appropriate comparators, predefined endpoints, sufficient statistical power, and appropriate safety monitoring [175]. Moreover, the preparation evaluated in the clinical study should be representative of the proposed commercial ingredient because changes in peptide composition, processing, purification, or formulation may limit the applicability of clinical findings to the marketed product.

#### 5.12.3. Industrial Scalability, Economic Feasibility and Quality Control

Industrial translation depends not only on biological activity but also on the ability to manufacture peptide-rich preparations reproducibly and at an economically acceptable cost. Major cost drivers include raw-material availability, protein-extraction yield, enzyme price and enzyme-to-substrate ratio, hydrolysis time, energy and water consumption, membrane processing, chromatographic purification, concentration, drying, and analytical quality control [89,90,91,92,93,94,104,105,106,107,108].

Extensive purification of individual peptides may substantially increase production costs and reduce overall product yield. For food and nutraceutical applications, standardized peptide-rich hydrolysates may therefore be more economically scalable than highly purified individual peptides, provided that efficacy, safety, compositional consistency, and batch-to-batch reproducibility can be demonstrated for the complete preparation [104,105,106,107,108].

Batch-to-batch consistency represents an additional challenge because cultivar, geographical origin, environmental conditions, storage, protein-extraction procedures, and hydrolysis conditions may influence peptide composition. Quality-control specifications should therefore include, where appropriate, total protein and peptide content, molecular-weight distribution, degree of hydrolysis, chromatographic or LC–MS peptide fingerprints, selected marker peptides, moisture content, residual processing aids or enzymes, microbiological limits, contaminants, antinutritional factors, and storage stability [89,90,91,92,93,94,104,105,106,107,108]. Establishing validated acceptance ranges for these parameters is essential to ensure that the material used in experimental studies remains representative of commercial-scale production.

#### 5.12.4. Commercial and Regulatory Examples

The number of plant-derived peptide-rich ingredients that have progressed from experimental discovery to commercial application with publicly documented regulatory information remains limited. One notable example is PeptiStrong^®^, a commercially available peptide-rich ingredient derived from enzymatically hydrolyzed fava bean (*Vicia faba* L.) protein [179]. The corresponding fava bean protein hydrolysate was submitted to the U.S. FDA as GRAS Notice No. 1166. In November 2024, the FDA issued a “no questions” letter regarding the notifier’s GRAS conclusion for specified food applications at a proposed use level of 2.4 g per serving [178]. Importantly, this regulatory outcome concerns the safety of the intended food uses and should not be interpreted as FDA approval of efficacy or disease-related health claims.

Additional regulatory precedents include rice protein hydrolysate under GRAS Notice No. 944 [180] and barley and rice protein hydrolysate under GRAS Notice No. 1031 [181], for which the FDA also issued “no questions” letters concerning the respective GRAS conclusions for specified food uses. These examples demonstrate that plant protein hydrolysates can progress through established food-ingredient safety pathways while also illustrating the distinction between regulatory safety status and substantiation of biological efficacy or health-related claims.

Overall, the limited number of commercially advanced plant peptide ingredients with both transparent regulatory documentation and robust human evidence highlights an important translational gap. Future commercialization will require the parallel development of scalable manufacturing processes, compositional standardization, quality-control specifications, safety documentation, clinically relevant efficacy data, and regulatory strategies tailored to the intended jurisdiction, product category, conditions of use, and proposed claims.

## 6. Future Perspectives and Research Priorities

Future research on plant-derived bioactive peptides should prioritize rigorous sequence-level identification, standardized experimental validation, and improved reproducibility. Particular emphasis should be placed on confirming peptide identity and purity, reporting quantitative biological activity, including experimentally validated negative or weakly active sequences, and harmonizing gastrointestinal digestion, biochemical, cellular, and safety-assessment protocols [10,100,104,109,110,111,112,115,116,117,118,119,120].

Artificial intelligence and machine learning are expected to play an increasing role in peptide discovery by supporting bioactivity prediction, toxicity and allergenicity screening, candidate prioritization, and de novo sequence design [121,122,167,168,169,170]. Their reliability will depend on high-quality plant-specific datasets and iterative validation workflows in which computationally selected peptides are experimentally tested and the resulting positive and negative data are used to refine predictive models. Emerging technologies, including high-resolution peptidomics, ion-mobility mass spectrometry, multi-omics approaches, organoids, and advanced cellular models, may further improve mechanistic and translational assessment [10,109,110,111,112,113,119,167,168,169,170,171,172].

Sustainability and clinical translation should be addressed in parallel with bioactivity. Future studies should evaluate scalable and resource-efficient production from plant proteins and agro-industrial side streams, together with batch-to-batch consistency, quality control, and economically feasible purification strategies [17,89,90,91,92,93,94,104,105,106,107,108]. For candidates intended for health-related applications, gastrointestinal stability, intestinal transport, systemic stability, pharmacokinetics, safety, dose–response relationships, and appropriately designed animal and human studies will be essential for establishing physiological relevance and supporting regulatory translation [20,21,22,61,62,63,64,118,140,171,172].

## 7. Conclusions

Plant proteins combine two important but distinct attributes: protein nutritional quality and the capacity to release biologically active peptides. A favorable essential amino acid profile, high digestibility, or a high protein-quality score contributes to nutritional adequacy but does not by itself predict the biological activity of peptides generated from the parent protein. Peptide bioactivity depends instead on the primary protein sequence, accessibility of proteolytic cleavage sites, processing and hydrolysis conditions, and the sequence, residue position, conformation, physicochemical properties, stability, and target interactions of the released peptides [1,2,3,14,18].

Accordingly, the biological potential of plant-derived peptides should be interpreted through an evidence-based sequence extending from parent-protein characterization and controlled peptide release to high-confidence sequence identification and direct experimental validation. Putative peptide assignments and computational predictions are valuable for candidate prioritization but should not be considered evidence of efficacy in the absence of sequence-resolved analytical confirmation and testing of purified or synthesized peptides. Quantitative biochemical and cellular assays, gastrointestinal stability studies, intestinal transport models, animal experiments, and, where appropriate, human investigations provide progressively stronger evidence of physiological relevance [10,20,21,22,104,109,118,119].

An integrated in silico → in vitro → in vivo framework provides a useful approach to plant-peptide discovery and validation. Computational methods, including molecular docking, molecular dynamics, bioinformatics, and AI/ML, can prioritize candidates and generate mechanistic hypotheses [10,162,167,168,169]. Appropriately designed in vitro studies can provide evidence of sequence-specific activity, possible mechanisms, stability, transport, and selected safety endpoints [20,21,118]. Animal studies, where justified, can further assess physiological effects, while claims of efficacy in humans require appropriately designed human studies. Results from each stage can inform subsequent candidate selection and experimental design, making peptide discovery an iterative process [168,170].

The translational potential of plant-derived peptides will therefore depend not on individual structural descriptors, predicted activity, or isolated in vitro endpoints, but on reproducible progression across these levels of evidence. Future research should prioritize experimentally confirmed peptide sequences, standardized quantitative assays, gastrointestinal and systemic stability, intestinal transport, pharmacokinetics, safety, dose–response relationships, and physiologically relevant in vivo and human validation. Such an integrated approach can more reliably identify plant-derived peptides with genuine potential for functional foods, nutraceuticals, and other health-related applications while maintaining a clear distinction between the nutritional value of the parent protein and the biological activity of its derived peptides. By explicitly separating protein nutritional quality from peptide bioactivity and linking peptide discovery to progressively stronger levels of experimental and translational evidence, this review provides a framework that complements existing general reviews of plant-derived bioactive peptides.

## Figures and Tables

**Table 2:** Common enzymes and microbial systems used for plant protein hydrolysis, representative operating conditions, industrial availability, and relevance to bioactive peptide generation.

Enzyme /System	Main Cleavage Preference or Role	Typical Application	Representative Optimal/Working Conditions (pH, Temperature) **	Industrial Availability	Relevance to Bioactive Peptides	Ref.
Alcalase	Broad-specificity serine endoprotease	Extensive hydrolysis of legume, cereal, pseudocereal, and oilseed proteins	pH~7.0; ~55 °C	Widely available commercial food-grade protease	Generates diverse low-molecular-weight peptide mixtures and is widely used for producing potentially bioactive plant protein hydrolysates	[6,14,16,66,94,97]
Trypsin	Preferentially cleaves peptide bonds at the C-terminal side of Lys and Arg	Controlled hydrolysis and simulated intestinal digestion	pH~8.0; ~37 °C	Commercially available, although generally less economical than broad-specificity microbial proteases for bulk hydrolysis	Produces relatively predictable peptide fragments with basic C-terminal residues and facilitates LC–MS/MS-based identification	[6,19,31,94,98]
Chymotrypsin	Preferentially cleaves peptide bonds adjacent to aromatic residues, particularly Phe, Tyr, and Trp	Controlled hydrolysis and simulated gastrointestinal digestion	pH~8.0; ~37 °C	Commercially available, mainly for laboratory and specialized processing applications	May release hydrophobic and aromatic peptide sequences associated with antioxidant or ACE-inhibitory potential	[6,26,31,94,99]
Pepsin	Acid-active endoprotease with preference for bonds involving hydrophobic or aromatic residues	Simulated gastric digestion	pH~3.0; ~37 °C	Commercially available	Produces digestion-relevant peptide fragments and enables evaluation of peptide release under gastric conditions	[6,19,94,100]
Pancreatin	Mixture of pancreatic digestive enzymes, including proteases and peptidases	Simulated intestinal digestion	pH~7.0; ~37 °C	Commercially available as standardized digestive enzyme preparations	Mimics multienzyme peptide release during the intestinal phase of gastrointestinal digestion	[6,19,94,100]
Papain	Broad-specificity cysteine endoprotease	Food-grade hydrolysis of plant proteins	pH~5–9; ~50–65 °C	Widely available for food and industrial applications	Produces heterogeneous peptide mixtures under relatively mild reaction conditions	[6,14,16,19,94,101]
Bromelain	Broad proteolytic activity involving cysteine proteases	Hydrolysis of plant protein isolates and processing by-products	pH~6–7; ~50–60 °C	Widely available for food and industrial applications	Can generate low-molecular-weight peptide fractions with potential antioxidant or ACE-inhibitory activity	[6,14,16,19,94,102]
Flavourzyme	Commercial enzyme preparation with endoprotease and exopeptidase activities	Extensive hydrolysis, peptide modification, and debittering	pH~6.5; ~50 °C	Widely available as a commercial food-grade enzyme preparation	May remove terminal hydrophobic residues, increase the degree of hydrolysis, and reduce bitterness	[6,56,67,94,97]
Thermolysin	Preferentially cleaves peptide bonds on the N-terminal side of hydrophobic residues	Targeted production of hydrophobic peptide sequences	pH~7.5; ~65 °C	Commercial food-enzyme preparations are available	May favor the generation of hydrophobic sequences associated with ACE-inhibitory activity	[6,14,19,31,94,103]
Lactic acid bacteria	Strain-dependent cell-envelope proteinases and intracellular peptidases	Fermentation of cereals, legumes, and pseudocereals	Typically pH~4–6.5; ~25–42 °C; strain-dependent	Food-grade starter cultures are widely used industrially	Promote gradual protein degradation and peptide release and may improve digestibility and reduce selected antinutritional factors	[57,70,87,96]
*Bacillus* spp.	Production of extracellular proteases and peptidases	Submerged or solid-state fermentation and direct protein hydrolysis	Typically pH~6.5–9; ~30–50 °C; strain-dependent	Industrial strains and protease preparations are available	Generate extensive and compositionally diverse peptide mixtures	[57,70,87,96]
Filamentous fungi	Complex extracellular endoprotease and exopeptidase systems	Solid-state or submerged fermentation of plant materials	Typically pH~4–7; ~25–35 °C; species- and strain-dependent	Established industrial fermentation systems are available	Modify plant matrices, increase protein accessibility, and generate diverse peptide populations	[57,70,87,96]

** The listed pH and temperature values represent typical working or near-optimal ranges. Actual optimal conditions depend on enzyme source or commercial formulation, plant substrate, enzyme-to-substrate ratio, reaction time, ionic strength, and the desired degree of hydrolysis.

**Table 3:** Main techniques used for protein extraction, peptide generation, fractionation, purification, and structural characterization of plant-derived peptides.

Technique/ Method	Primary Purpose	Key Advantages	Major Limitations	References
Alkaline extraction	Solubilization of plant storage proteins	High protein recovery; simple and scalable	Harsh pH may induce denaturation, oxidation, deamidation, aggregation, or amino acid modification	[14,16,17,89,90]
Isoelectric precipitation	Recovery and concentration of solubilized proteins	Widely used, cost-effective, and scalable	May reduce protein solubility and alter techno-functional properties	[14,16,17,89,90]
Salt-based extraction	Mild extraction of albumins and globulins	Preserves protein conformation under relatively mild and food-compatible conditions	May provide lower recovery for some plant matrices and requires subsequent salt removal	[14,16,30,89,90]
Enzyme-assisted extraction	Degradation of cell-wall polysaccharides and enhancement of protein release	Mild and selective; may improve protein accessibility and recovery	Enzyme cost, reaction time, and process optimization requirements	[59,60,90,92]
Ultrasound-assisted extraction	Cell disruption and enhancement of mass transfer	Shorter extraction time and reduced solvent consumption	Excessive cavitation may promote oxidation, aggregation, or structural modification	[59,60,92,93]
Microwave-assisted extraction	Rapid heating and disruption of the plant matrix	Fast and efficient extraction	Excessive thermal exposure may induce denaturation or aggregation	[59,60,92]
High-pressure processing	Modification of cellular structures and improvement of protein extractability	Non-thermal or low-thermal treatment; may improve protein functionality	High equipment and operating costs; effects are matrix-dependent	[59,60,92]
Pulsed electric fields	Cell membrane permeabilization and enhancement of intracellular protein release	Energy-efficient and generally non-thermal	Efficiency depends strongly on matrix conductivity, electric-field strength, and treatment conditions	[59,60,92,93]
Enzymatic hydrolysis	Controlled release of peptides from plant proteins	Selective, reproducible, and compatible with mild processing conditions	Enzyme cost; bitterness or excessive hydrolysis may occur	[6,14,16,19,31,55,58,65,94,95]
Microbial fermentation	Proteolysis and generation of potentially bioactive peptide mixtures	May reduce selected antinutritional factors and improve digestibility and sensory properties	Peptide profiles are complex, strain-dependent, and difficult to standardize	[57,70,87,96]
Germination	Activation of endogenous proteases and mobilization of storage proteins	Natural, relatively low-cost, and potentially beneficial for nutritional quality	Strong variability among species, cultivars, germination stages, and processing conditions	[16,25,45,57,70]
Ultrafiltration	Approximate molecular-size-based fractionation of peptide mixtures	Scalable and compatible with food-grade processing	Membrane fouling, concentration polarization, and limited resolution between similarly sized peptides	[8,105,106,107]
RP-HPLC	Hydrophobicity-based purification of peptide fractions	High resolution and direct compatibility with mass spectrometry	Organic-solvent consumption, relatively low throughput, and limited scalability	[8,104,105,106,107,108]
Ion-exchange chromatography	Charge-based separation of peptide populations	Effective separation of acidic, neutral, and basic peptides	Salt gradients may require desalting before downstream MS analysis	[104,105,106,107,108]
Size-exclusion chromatography	Separation and profiling according to hydrodynamic size	Gentle separation under mild conditions	Limited resolution for peptides with similar molecular sizes	[104,105,106,107,108]
HILIC	Separation of highly polar and hydrophilic peptides	Complementary to reversed-phase chromatography	Method development may be complex, and retention is sensitive to mobile-phase composition	[104,105]
LC–MS/MS	Peptide separation, fragmentation, sequencing, and identification	Sensitive, accurate, and suitable for complex peptide mixtures	Requires advanced instrumentation, extensive data processing, and reliable databases	[108,109,112]
MALDI-TOF MS	Rapid peptide mass profiling	Fast, robust, and useful for preliminary screening	Provides limited sequence information unless coupled with tandem MS	[8,104,108,109]
Orbitrap or Q-TOF MS	High-resolution peptide identification and accurate mass measurement	High mass accuracy, sensitivity, and improved sequence confidence	Expensive instrumentation, maintenance, and specialized expertise	[104,109]
Ion mobility–mass spectrometry	Gas-phase separation of structurally or conformationally distinct peptide ions	Provides collision cross-section information and an additional separation dimension	Requires specialized instrumentation, software, and careful interpretation	[104,113]
Circular dichroism spectroscopy	Estimation of peptide secondary-structure populations in solution	Rapid and non-destructive conformational assessment	Requires relatively pure samples and provides ensemble-averaged structural information	[114,123]
FTIR spectroscopy	Analysis of peptide backbone conformation through amide bands	Rapid and useful for monitoring secondary-structure-related changes	Band overlap and sample conditions may limit structural resolution	[114,124]
NMR spectroscopy	Determination of three-dimensional structure, conformational dynamics, and molecular interactions	Provides residue-level structural and conformational information	Requires purified peptides, relatively high sample amounts, and substantial acquisition and analysis time	[114,125]
Fluorescence spectroscopy	Investigation of peptide binding and conformational changes	Highly sensitive and suitable for monitoring molecular interactions	Often depends on intrinsic fluorescent residues or external probes and may be affected by inner-filter effects	[114,126]

**Table 4:** Representative sequence-resolved plant-derived bioactive peptides with direct experimental validation and corresponding levels of evidence.

Plant Source	Peptide Sequence	Biological Activity	Experimental Model/ Key Finding	Level of Evidence	Ref.
*Paeonia ostii* ‘Feng Dan’ seed meal	EAAY (Glu-Ala-Ala-Tyr)	Antioxidant	Identified by nano-LC–MS/MS; showed 98.5 ± 1.1% ABTS radical-scavenging activity and 61.9 ± 1.3% hydroxyl radical-scavenging activity at 0.5 mg/mL. No obvious cytotoxicity was observed toward L929 fibroblast cells	Biochemical activity + cellular safety assessment; supported by in silico analysis	[18]
Soybean (*Glycine max*) protein isolate	NDRP (Asn-Asp-Arg-Pro)	ACE inhibitory	Initially identified among four putative sequences and subsequently synthesized by solid-phase synthesis. NDRP showed 87.76 ± 0.61% ACE inhibition with IC_50_ = 148.28 ± 9.83 μg/mL, acted as a non-competitive inhibitor, remained stable during simulated gastrointestinal digestion, and was confirmed in the hydrolysate by MRM	Biochemical + gastrointestinal stability + targeted sequence confirmation	[127]
*Vicia faba*-derived hydrolysate (NPN_1)	HLPSYSPSPQ	Protein synthesis-promoting	Initially predicted by machine learning and subsequently validated in vitro for stimulation of protein synthesis. The peptide survived upper gastrointestinal digestion, crossed an intestinal barrier model, and showed notable stability in human plasma	Computational + cellular + gastrointestinal stability + intestinal transport + plasma stability	[21]
*Vicia faba*-derived hydrolysate (NPN_1)	TIKIPAGT	Anti-inflammatory	Initially predicted by machine learning and subsequently validated in vitro by reduction in TNF-α secretion. The peptide survived upper gastrointestinal digestion, crossed an intestinal barrier model, and showed notable stability in human plasma	Computational + cellular + gastrointestinal stability + intestinal transport + plasma stability	[21]
Fermented soybean (Douchi)	VY (Val-Tyr)	Hypoglycemic/glucose-regulatory	Resistant to simulated gastrointestinal digestion and transported intact across Caco-2 monolayers through paracellular and PepT1-mediated pathways. In HFD-induced insulin-resistant C57BL/6 mice, 10 or 50 mg/kg for 8 weeks improved glucose homeostasis and insulin sensitivity	Cellular/function-related + gastrointestinal stability + intestinal transport + animal	[20,128]
Fermented soybean (Douchi)	SFLLR (Ser-Phe-Leu-Leu-Arg)	Hypoglycemic/glucose-regulatory	Partially digested to SFLL, SFL, and SF, with increased glucose-uptake activity; transported across Caco-2 monolayers. In HFD-induced insulin-resistant mice, 10 or 50 mg/kg for 8 weeks, particularly SFLLR, reduced insulin resistance and hyperglycemia	Cellular/function-related + gastrointestinal digestion + intestinal transport + animal	[20,128]
Soybean (*Glycine max*)	Lunasin (43-aa peptide)	Reported anti-inflammatory/anticancer-related peptide; human bioavailability	In five healthy men consuming 50 g soy protein/day for 5 days, plasma lunasin averaged 66.0 ± 25.4 ng/mL at 30 min and 71.0 ± 32.8 ng/mL at 1 h; estimated absorption averaged 4.5%. MALDI-TOF and LC–MS/MS detected lunasin-related sequences in post-intake plasma	Human bioavailability/absorption; not clinical efficacy	[22]

**Table 5:** Representative computational databases and tools used for the identification, prediction, and molecular evaluation of plant-derived bioactive peptides.

Database/Tool	Primary Function	Computational Approach	Major Limitation	Recommended Experimental Validation	References
BIOPEP-UWM	Database of experimentally validated bioactive peptides; in silico enzymatic digestion and peptide profiling	Database matching, occurrence-frequency analysis, enzyme-specific cleavage simulation	Rule-based digestion does not account for protein conformation, cleavage-site accessibility, or digestion kinetics	Confirm predicted peptide release by controlled enzymatic hydrolysis or simulated digestion followed by LC–MS/MS sequence identification	[152]
PeptideRanker	General prediction of peptide bioactivity	Machine-learning model based on sequence-derived features	Predicts general bioactivity but not a specific biological function	Chemically synthesize or purify high-ranking peptides and evaluate them in concentration-dependent activity assays relevant to the proposed function	[150]
CAMPR4	Prediction and annotation of antimicrobial peptides	Support vector machine, random forest, artificial neural network, and discriminant analysis	Designed specifically for antimicrobial peptides; does not predict other peptide activities	Determine MIC and, where appropriate, MBC values using standardized antimicrobial assays; additionally assess membrane effects and mammalian-cell cytotoxicity	[154]
DBAASP v3	Database of antimicrobial and cytotoxic peptides	Curated experimental database with sequence, structural, and activity information	Activity values originate from heterogeneous experimental conditions	Compare candidate peptides under standardized antimicrobial, hemolysis, and cytotoxicity conditions rather than relying directly on heterogeneous database activity values	[155]
ToxinPred 3.0	Prediction of peptide toxicity	Machine-learning models using sequence composition and physicochemical descriptors	Computational prediction cannot replace experimental cytotoxicity testing	Perform dose-dependent cytotoxicity testing in relevant mammalian cell lines and, where justified, additional toxicological evaluation	[156]
AllerTOP v2.0	Prediction of allergenic potential	Alignment-free k-nearest-neighbor classifier based on amino acid descriptors	Lower reliability for short peptides and chemically modified sequences	Follow computational screening with allergenicity assessment using sequence homology analysis, IgE-binding assays, basophil-activation tests, or other appropriate in vitro or in vivo models	[157]
HemoPI https://webs.iiitd.edu.in/raghava/hemopi/	Prediction of peptide hemolytic activity	Support vector machine models using sequence-derived features	Does not quantify hemolysis under specific experimental conditions	Perform concentration-dependent erythrocyte hemolysis assays and determine hemolytic selectivity relative to the intended biological activity	[158]
ExPASy ProtParam	Calculation of physicochemical properties	Sequence-based calculation of molecular weight, theoretical pI, instability index, aliphatic index, and GRAVY	Does not consider three-dimensional structure or biological environment	Experimentally confirm properties relevant to application, particularly peptide identity, solubility, aggregation, chemical stability, and stability in biological media	[159]
AutoDock Vina	Molecular docking of peptide–target complexes	Empirical scoring function with global and local conformational search	Limited treatment of peptide flexibility, solvent effects, and receptor dynamics	Validate predicted interactions using concentration-dependent enzyme inhibition, kinetic analysis, direct binding measurements, or residue-substitution studies with purified proteins or target biomolecules	[160]
GROMACS	Molecular dynamics simulation of peptide–target interactions	Force-field-based atomistic molecular dynamics simulations	Results depend on force field, simulation time, and sampling strategy	Test predicted interaction stability through biochemical or biophysical binding assays, mutational or peptide-substitution studies, and functional assays; for membrane-active peptides, use membrane-permeabilization or related biophysical assays	[161]

## Data Availability

No new data were created or analyzed in this study. Data sharing is not applicable to this article.

## Abbreviations

The following abbreviations are used in this manuscript:

ABTS	2,2′-Azino-bis(3-ethylbenzothiazoline-6-sulfonic acid)
ACE	Angiotensin-Converting Enzyme
AI	Artificial Intelligence
AMP	Antimicrobial Peptide
CAT	Catalase
CD	Circular Dichroism
CID	Collision-Induced Dissociation
COX-2	Cyclooxygenase-2
DPPH	2,2-Diphenyl-1-picrylhydrazyl
DPP-IV	Dipeptidyl Peptidase-IV
eNOS	Endothelial Nitric Oxide Synthase
ERK	Extracellular Signal-Regulated Kinase
ESI	Electrospray Ionization
ETD	Electron-Transfer Dissociation
FDR	False Discovery Rate
FRAP	Ferric Reducing Antioxidant Power
FTIR	Fourier-Transform Infrared Spectroscopy
GLP-1	Glucagon-Like Peptide-1
GIP	Glucose-Dependent Insulinotropic Polypeptide
GPx	Glutathione Peroxidase
HCD	Higher-Energy Collisional Dissociation
HILIC	Hydrophilic Interaction Liquid Chromatography
HO-1	Heme Oxygenase-1
HPLC	High-Performance Liquid Chromatography
IC50	Half-Maximal Inhibitory Concentration
IL	Interleukin
iNOS	Inducible Nitric Oxide Synthase
JNK	c-Jun N-terminal Kinase
LC-MS/MS	Liquid Chromatography–Tandem Mass Spectrometry
MALDI-TOF MS	Matrix-Assisted Laser Desorption/Ionization Time-of-Flight Mass Spectrometry
MAPK	Mitogen-Activated Protein Kinase
MIC	Minimum Inhibitory Concentration
MM-GBSA	Molecular Mechanics Generalized Born Surface Area
MM-PBSA	Molecular Mechanics Poisson–Boltzmann Surface Area
MS	Mass Spectrometry
MW	Molecular Weight
NF-κB	Nuclear Factor Kappa B
NMR	Nuclear Magnetic Resonance
Nrf2	Nuclear Factor Erythroid 2-Related Factor 2
ORAC	Oxygen Radical Absorbance Capacity
PCA	Principal Component Analysis
PepT1	Peptide Transporter 1
PEF	Pulsed Electric Field
PGE2	Prostaglandin E2
Q-TOF	Quadrupole Time-of-Flight
RAAS	Renin–Angiotensin–Aldosterone System
RNS	Reactive Nitrogen Species
ROS	Reactive Oxygen Species
RP-HPLC	Reversed-Phase High-Performance Liquid Chromatography
SOD	Superoxide Dismutase
TNBS	Trinitrobenzenesulfonic Acid
TNF-α	Tumor Necrosis Factor Alpha
